# BRCA1 degradation in response to mitochondrial damage in breast cancer cells

**DOI:** 10.1038/s41598-021-87698-7

**Published:** 2021-04-22

**Authors:** Kana Miyahara, Naoharu Takano, Yumiko Yamada, Hiromi Kazama, Mayumi Tokuhisa, Hirotsugu Hino, Koji Fujita, Edward Barroga, Masaki Hiramoto, Hiroshi Handa, Masahiko Kuroda, Takashi Ishikawa, Keisuke Miyazawa

**Affiliations:** 1 Department of Breast Oncology and Surgery, Tokyo Medical University, Shinjuku, Tokyo 160-8402 Japan; 2 Department of Biochemistry, Tokyo Medical University, Shinjuku, Tokyo 160-8402 Japan; 3 Department of Molecular Pathology, Tokyo Medical University, Shinjuku, Tokyo 160-8402 Japan; 4 Department of Nanoparticle Translational Research, Tokyo Medical University, Shinjuku, Tokyo 160-8402 Japan; 5 St. Luke’s International University, Tokyo, 104-0044 Japan

**Keywords:** Biochemistry, Cell biology, Cancer, Breast cancer

## Abstract

BRCA1 is a well-studied tumor suppressor involved in the homologous repair of DNA damage, whereas PINK1, a mitochondrial serine/threonine kinase, is known to be involved in mitochondrial quality control. Genetic mutations of *PINK1* and *Parkin* cause autosomal recessive early-onset Parkinson’s disease. We found that in breast cancer cells, the mitochondrial targeting reagents, which all induce mitochondrial depolarization along with PINK1 upregulation, induced proteasomal BRCA1 degradation. This BRCA1 degradation was dependent on PINK1, and BRCA1 downregulation upon mitochondrial damage caused DNA double-strand breaks. BRCA1 degradation was mediated through the direct interaction with the E3 ligase Parkin. Strikingly, BRCA1 and PINK1/Parkin expression were inversely correlated in cancerous mammary glands from breast cancer patients. BRCA1 knockdown repressed cancer cell growth, and high *BRCA1* expression predicted poor relapse-free survival in breast cancer patients. These observations indicate a novel mechanism by which mitochondrial damage is transmitted to the nucleus, leading to BRCA1 degradation.

## Introduction

Breast cancer susceptibility gene 1 (BRCA1) is responsible for hereditary breast and ovarian cancers. The incidences of breast and ovarian cancers by 70 years of age in carriers of a BRCA1 mutation are 65% and 39%, respectively^1^. BRCA1 is involved in multiple cellular processes, including DNA repair, transcriptional regulation, cell cycle checkpoint, apoptosis, chromatin remodeling, and centrosome replication^2–4^. DNA double-strand break repair by homologous recombination (HR) is one of the primary mechanisms by which BRCA1 acts as a tumor suppressor. In tumors that lack BRCA1 function, elevated DNA instability confers sensitivity to poly (ADP-ribose) polymerase (PARP) inhibitors of single-strand break repair compensating for the lack of HR^5,6^. Reflecting on its diverse biological activities, BRCA1 consists of multiple domains: in order from its N-terminus, the RING, DNA binding, coiled-coil, and BRCA1 C-terminal (BRCT) domains. Through these domains, BRCA1 binds various proteins to exert its many functions^7^. BRCA1-associated RING domain 1 (BARD1) forms a heterodimer with BRCA1 through the two proteins’ RING domains^8^, masking the nuclear export signal of BRCA1 and keeping it in the nucleus. Several lines of evidence show that BRCA1 expression is regulated by the ubiquitin–proteasome system; this regulation involves several E3 ubiquitin ligases, including HERC2, HUWE1, and FBXO44^9–14^. In addition, BARD1 protects BRCA1 from ubiquitin–proteasome degradation by preventing HERC2 from binding the N-terminal degron domain in BRCA1, leading to a higher nuclear expression^13^.

*PINK1* (*PARK6*) and *Parkin* (*PRKN*, *PARK2*) are causal genes for autosomal recessive early-onset Parkinsonism and key components for mediating the quality control of mitochondria^15^. When mitochondria lose their membrane potential, triggering mitophagy, PTEN-induced putative kinase 1 (PINK1) is stabilized on the mitochondrial outer membrane (MOM); subsequently, PINK1 phosphorylates both the E3 ligase Parkin and ubiquitin^16–21^, which finally induces the ubiquitination of MOM proteins, and subsequently promotes the engulfment of the depolarized mitochondria by autophagosomes for the execution of mitophagy^22^. Defective mitophagy is thought to contribute to a variety of diseases, including cancer^23^.

We herein report that BRCA1 is degraded in response to loss of mitochondrial membrane potential. This proteasomal degradation is dependent on PINK1 and at least in part mediated through the E3 ligase Parkin. Additionally, the downregulation of BRCA1 repressed breast cancer cell growth. Our findings support a novel mechanism by which information about mitochondrial damage is passed to the nucleus, manifesting as BRCA1 degradation.

## Results

### Mitochondrial damage induces PINK1 upregulation leading to BRCA1 degradation, which is then followed by the induction of DNA double-strand breaks

**Figure 1:**
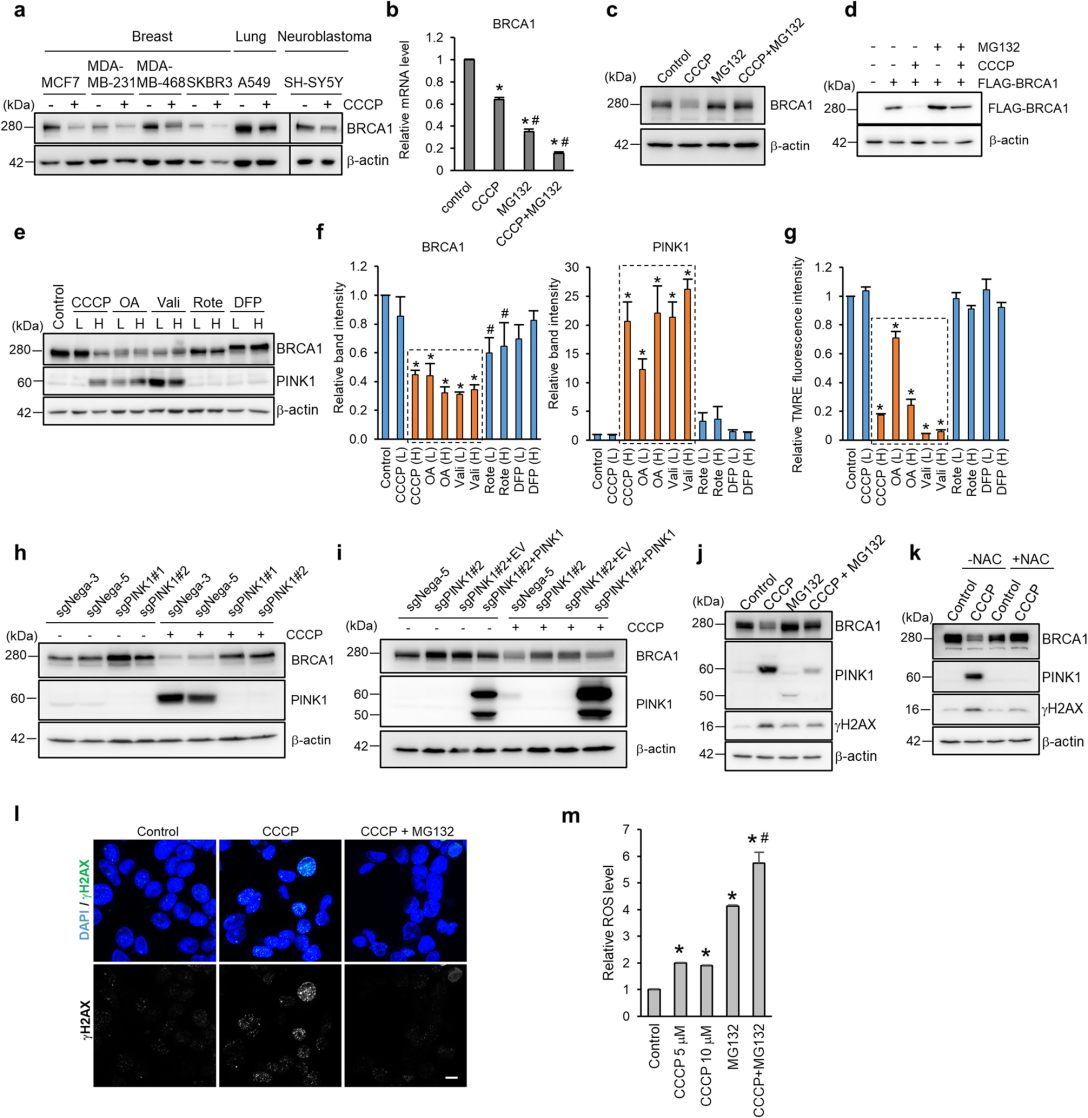
Mitochondrial damage promotes PINK1-dependent BRCA1 degradation. (**a**) Western blotting analysis of BRCA1 in indicated cell lines treated with or without 10 μM CCCP for 24 h. (**b**) BRCA1 mRNA expression level was assessed after treatment with CCCP ± 10 μM MG132 for 24 h. *n* = 3, bar = means ± S.E., *p < 0.01 versus control, ^#^*p* < 0.01 versus CCCP. (**c**) MCF7 cells were treated with the indicated agents for 24 h and BRCA1 protein level was assessed. (**d**) 293 T cells were transfected with FLAG-tagged BRCA1 and treated with the indicated agents for 24 h. (**e**) MCF7 cells were treated with CCCP (2 or 10 μM), oligomycin A and antimycin A (OA) (0.5 μM + 50 nM or 2.5 μM + 250 nM), valinomycin (Vali) (0.5 or 2 μM), rotenone (Rote) (0.5 or 2 μM), or DFP (0.2 or 1 mM) for 24 h. L and H respectively indicate low- and high-dose treatments. (**f**) The calculated band intensities of BRCA1 and PINK1 were shown. Bar = means ± S.E., *n* = 3. *p < 0.01, ^#^*p* < 0.05 versus control. (**g**) Mitochondrial membrane potential was measured. Bar = means ± S.E., *n* = 3, *p < 0.05 versus control. (**h**) PINK1 knockout MCF7 cells or control cells were treated with ± 10 μM CCCP for 24 h. (**i**) BRCA1 expression level in sgPINK1#2 cells expressing PINK1 or empty vector (EV) was assessed after CCCP treatment for 24 h. (**j**) MCF7 cells were treated with the indicated agents for 24 h and assessed DNA double-strand breaks by detecting γH2AX expression. (**k**) MCF7 cells were treated with CCCP ± 10 mM NAC. (**l**) Immunostaining of γH2AX in MCF7 cells treated with the indicated agents. Nuclei were stained with DAPI. γH2AX signals and nuclei are shown as green and blue, respectively. Scale bar = 10 μm. (**m**) ROS levels were assessed in MCF7 cells treated with the indicated agents for 24 h. *n* = 3, bar = means ± S.D. *p < 0.05 versus control. ^#^*p* < 0.05 versus MG132.

We observed that treatment of MCF7 cells with CCCP, a mitochondrial uncoupling agent, induced the downregulation of BRCA1. A decrease of BRCA1 expression in response to CCCP treatment was observed in various breast cancer cell lines tested as well as in lung cancer and a neuroblastoma cell line, all of which express wild-type BRCA1 (Fig. 1a). Real-time PCR revealed that CCCP treatment repressed *BRCA1* mRNA to some extent, but the downregulation of BRCA1 protein was almost completely abolished in the presence of MG132, a proteasome inhibitor (Fig. 1b,c). Additionally, the exogenously expressed FLAG-BRCA1, which was expressed under CMV-promoter, in HEK293T cells was completely abolished by CCCP treatment, and this abolishment was cancelled by the co-administration of MG132 (Fig. 1d). Thus, the BRCA1 downregulation was mainly mediated through proteasomal degradation and was not due to transcriptional modification. Since PINK1, a serine/threonine kinase stabilized on the mitochondrial outer membrane (MOM), coincides with a decreased mitochondrial membrane potential^17,18^, we next focused on PINK1 in the context of BRCA1 downregulation following mitochondrial damage. We treated MCF7 cells with various mitochondria-targeted agents that induce mitophagy at different doses (low or high). These agents included oligomycin A (ATP synthase inhibitor), antimycin A (complex III inhibitor), valinomycin (K^+^ ionophore), rotenone (complex I inhibitor), and deferiprone (DFP, iron chelator). We then assessed the expression of PINK1 and BRCA1 in the treated cells (Fig. 1e,f). Treatment with CCCP at a high concentration, a combination of oligomycin A and antimycin A (OA) at high and low concentrations, or valinomycin at high and low concentrations all increased PINK1 expression and decreased BRCA1 expression. On the other hand, rotenone weakly increased PINK1 expression and only slightly decreased BRCA1 expression, and DFP had no effect on the level of either protein (Fig. 1f). We also assessed the mitochondrial membrane potential of the treated cells (Fig. 1g). Except for the low dose of CCCP, both doses of DFP, and both doses of rotenone, all other agents decreased the mitochondrial membrane potential. This indicates the strong correlation between BRCA1 downregulation and PINK1 upregulation upon a reduced mitochondrial membrane potential. To clarify the involvement of PINK1 in BRCA1 degradation, we established PINK1 knockout MCF7 clones with the CRISPR-Cas9 system using 2 different guide RNAs (sgPINK1#1 and sgPINK1#2). As we expected, PINK1 knockout increased BRCA1 expression and attenuated the reduction of the BRCA1 level after treatment with CCCP (Fig. 1h). Additionally, re-expression of PINK1 rescued BRCA1 degradation after CCCP treatment (Fig. 1i). All these data indicate that BRCA1 degradation is regulated by PINK1. BRCA1 expression level has been reported to be regulated by cell cycle on mRNA and protein level, which leads to higher BRCA1 expression in S to G2/M phase^13,24,25^. Thus, we assessed the cell cycle before and after CCCP treatment in control and PINK1 knockout MCF7 cells to verify whether the BRCA1 downregulation depends on the cell cycle. Since both cell lines exhibited almost equivalent increment of the cells in G1/G0 phase upon CCCP treatment, the influence of cell cycle in BRCA1 downregulation appeared to be small if any in this case (Fig. S1). To further assess whether the PINK1-dependent BRCA1 expression is cell type-specific, we established PINK1-KO MDA-MB-231 and MDA-MB-468 cells and assessed BRCA1 expression level before and after the CCCP treatment. Consistent with the results in MCF7 cells, PINK1-KO increased the basal expression level of BRCA1 in MDA-MB-468 cells but not in MDA-MB-231 cells, and the CCCP-induced BRCA1 degradation was not attenuated (Fig. S2), suggesting that this PINK1-dependent BRCA1 degradation may be cell type- or context-dependent. As PINK1-dependent BRCA1 degradation in MCF7 cells was firmly reproducible, we have attempted to verify the connection between PINK1 and BRCA1 degradation in MCF7 cells.

Given that BRCA1 repairs DNA damage via HR, we also assessed DNA damage following CCCP treatment. We observed elevated levels of γH2AX, which accumulates at sites of double-strand breaks in nuclear genomic DNA^26^, as well as BRCA1 degradation following CCCP treatment (Fig. 1j–l). In the presence of MG132, γH2AX induction was suppressed and BRCA1 degradation was blocked. This indicates that DNA double-strand breaks might be more prevalent following CCCP treatment owing to BRCA1 downregulation.

Impaired mitochondria produce reactive oxygen species (ROS), which subsequently induce DNA damage^27^. As shown in Fig. 1k, CCCP-induced BRCA1 degradation and PINK1 upregulation, as well as accumulation of γH2AX, were all suppressed in the presence of NAC. To address whether an increase in γH2AX expression is a direct effect of ROS or is instead due to the machinery of the PINK1 upregulation, we compared ROS production between CCCP-treated and MG132-treated MCF7 cells (Fig. 1m). Both CCCP and MG132 treatments significantly increased ROS production, but notably, co-treatment with CCCP plus MG132 suppressed γH2AX accumulation (Fig. 1j,l). This implies that the increase in γH2AX is not due to a direct effect of ROS, but rather due to the downregulation of BRCA1.

### Parkin overexpression enhanced CCCP-induced BRCA1 degradation and colocalize with BRCA1 in nucleus

**Figure 2:**
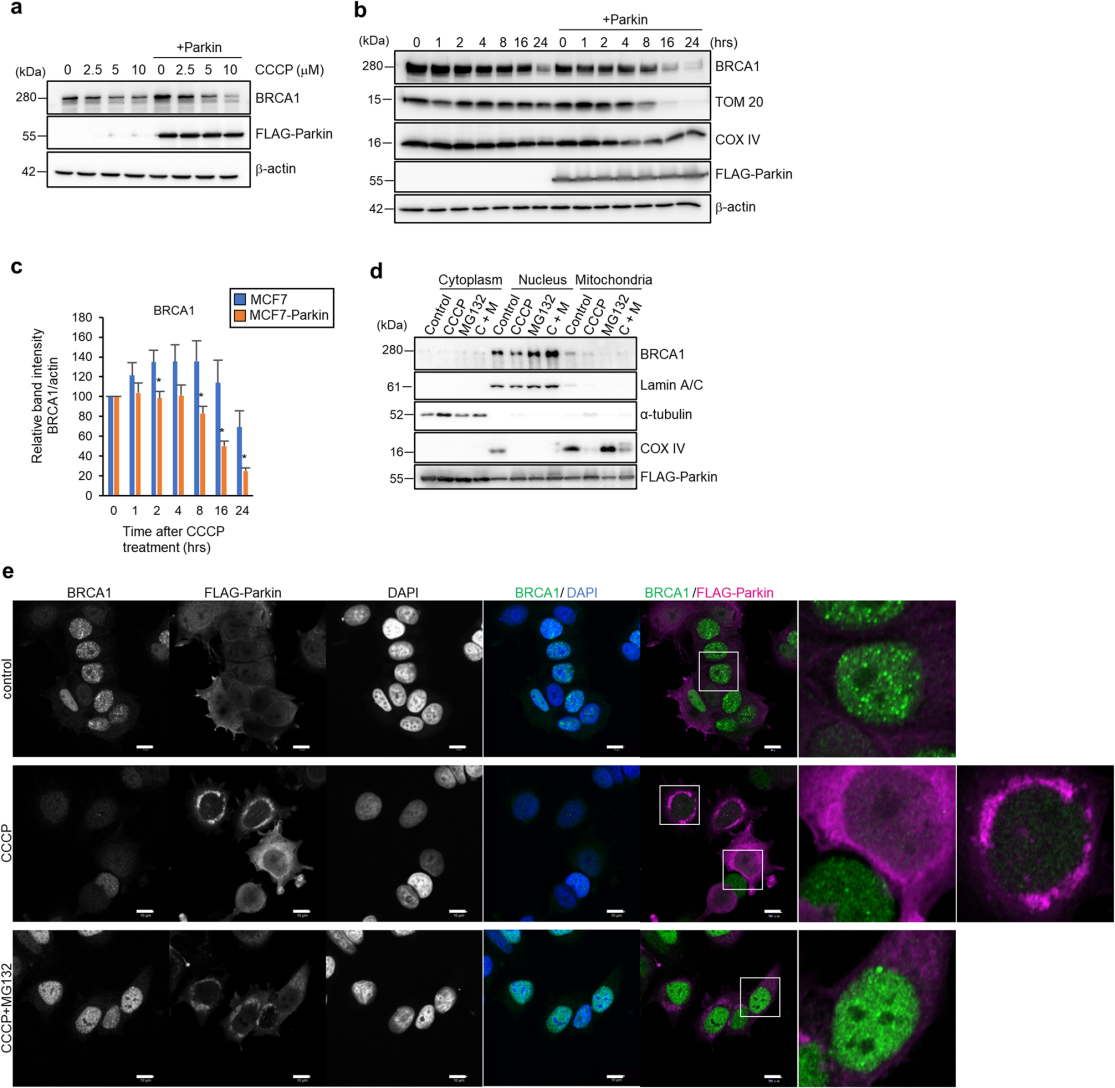
Parkin promotes BRCA1 degradation after mitochondrial damage via the ubiquitin–proteasome pathway and is co-localized in nucleus. (**a**) MCF7 and MCF7-Parkin cells were treated with CCCP at the indicated concentrations for 24 h, and then BRCA1 protein level was assessed by Western blotting. (**b**) Protein levels of BRCA1 as assessed by Western blotting. MCF7 and MCF7-Parkin cells were treated with 10 μM CCCP for the indicated times. Representative images from seven independent experiments are shown. TOM20 and COX IV were assessed to verify the occurrence of mitophagy. Because the overexpression of Parkin accelerates mitophagy, the degradation of TOM20 and COX IV in MCF7-Parkin cells after CCCP treatment was faster than that in MCF7 cells. (**c**) Densitometry analysis of BRCA1 in (**b**). Each band intensity was standardized against β-actin, and relative band intensities were calculated. *n* = 7, bar = means ± S.E., *p < 0.05 versus MCF7 at the same time point. (**d**) MCF7-Parkin cells were separated into the cytoplasmic, nuclear, and mitochondrial fractions after treatment with 0 or 10 µM CCCP and 0 or 10 µM MG132 for 24 h. Each fraction was subjected to Western blotting. Lamin A/C, α-tubulin, and COX IV were used as markers of the nuclear, cytoplasmic, and mitochondrial fractions, respectively. (**e**) Subcellular localizations of BRCA1 and FLAG-Parkin in MCF7-Parkin cells were assessed by immunofluorescence. Cells were treated with a vehicle, 10 μM CCCP, or 10 µM CCCP + 10 μM MG132 for 5 h. The treatments are indicated on the left side, and the detected proteins are indicated at the top. In the merged panels, BRCA1, FLAG-Parkin, and the nuclei are shown in green, magenta, and blue, respectively. The small boxed images are enlarged in the right panels. Scale bar = 10 μm.

It has been intensively studied and well known that depolarization of the mitochondrial membrane potential leads to stabilization and activation of PINK1 followed by activation of E3 ligase Parkin, which in turn ubiquitinates MOM proteins^22^. Because our data showed BRCA1 was degraded by the ubiquitin–proteasome system under PINK1 upregulation, we next focused on the role of Parkin, which is well known E3 ligase activated by PINK1, in BRCA1 degradation. The overexpression of Parkin in MCF7 cells resulted in pronounced CCCP-induced BRCA1 degradation in a dose- and time-dependent manner (Fig. 2a–c). This strongly indicates Parkin is an E3 ligase responsible for CCCP-induced BRCA1 degradation. Since Parkin overexpression enhanced mitophagy, to exclude the involvement of mitophagy on BRCA1 degradation, we co-administered bafilomycin A_1_ (BafA_1_) with CCCP in MCF7 cells or MCF7-Parkin cells (Fig. S3a). Although BafA1 inhibited lysosome-dependent degradation, BRCA1 degradation was not attenuated. Moreover, in ATG5-KO A549 cells, which lack autophagosome formation, CCCP administration degraded BRCA1 (Fig. S3b). These data indicated that BRCA1 degradation is independent of autophagy and/or mitophagy. Parkin accumulates on the MOM in response to mitochondrial damage^21^. A splice variant of BRCA1 localizes on the mitochondria, but most BRCA1 localizes in the nucleus^28^. To clarify the subcellular location where BRCA1 directly interacts with Parkin, we performed Western blotting of the fractionated cell lysates and immunostained FLAG-Parkin and BRCA1 in MCF7-Parkin cells following treatment with CCCP in the presence or absence of MG132 (Fig. 2d,e). The fractionation data revealed widespread FLAG-Parkin localization, a reduction in nuclear BRCA1 following CCCP treatment, and recovery of nuclear BRCA1 expression in the presence of MG132 (Fig. 2d). Immunostaining showed that in untreated cells, BRCA1 mainly localized in the nucleus, and only a faint cytosolic signal was detectable. The nuclear BRCA1 signal was reduced following CCCP treatment, but was completely recovered in the presence of MG132. In untreated control cells, FLAG-Parkin signals were distributed in the cytoplasm as well as the nucleus, although the cytoplasmic signal was denser, consistent with the results of immunoblotting (Fig. 2e). In response to CCCP treatment, the FLAG-Parkin signal accumulated at perinuclear sites, where mitochondria also localize, but was still detectable in the nucleus (Fig. 2e). Notably, the CCCP-induced downregulation of nuclear BRCA1 was restored or even enhanced in the presence of MG132. By contrast, we observed no increase in the cytoplasmic BRCA1 signal in the presence of MG132. Furthermore, cells with a stronger nuclear FLAG-Parkin signal contained a weaker nuclear BRCA1 signal, and vice versa (Fig. 2e, second row). Therefore, the main degradation site of BRCA1 appears to be the nucleus, rather than the cytoplasm.

### Parkin ubiquitinates BRCA1 via a direct intermolecular interaction

**Figure 3:**
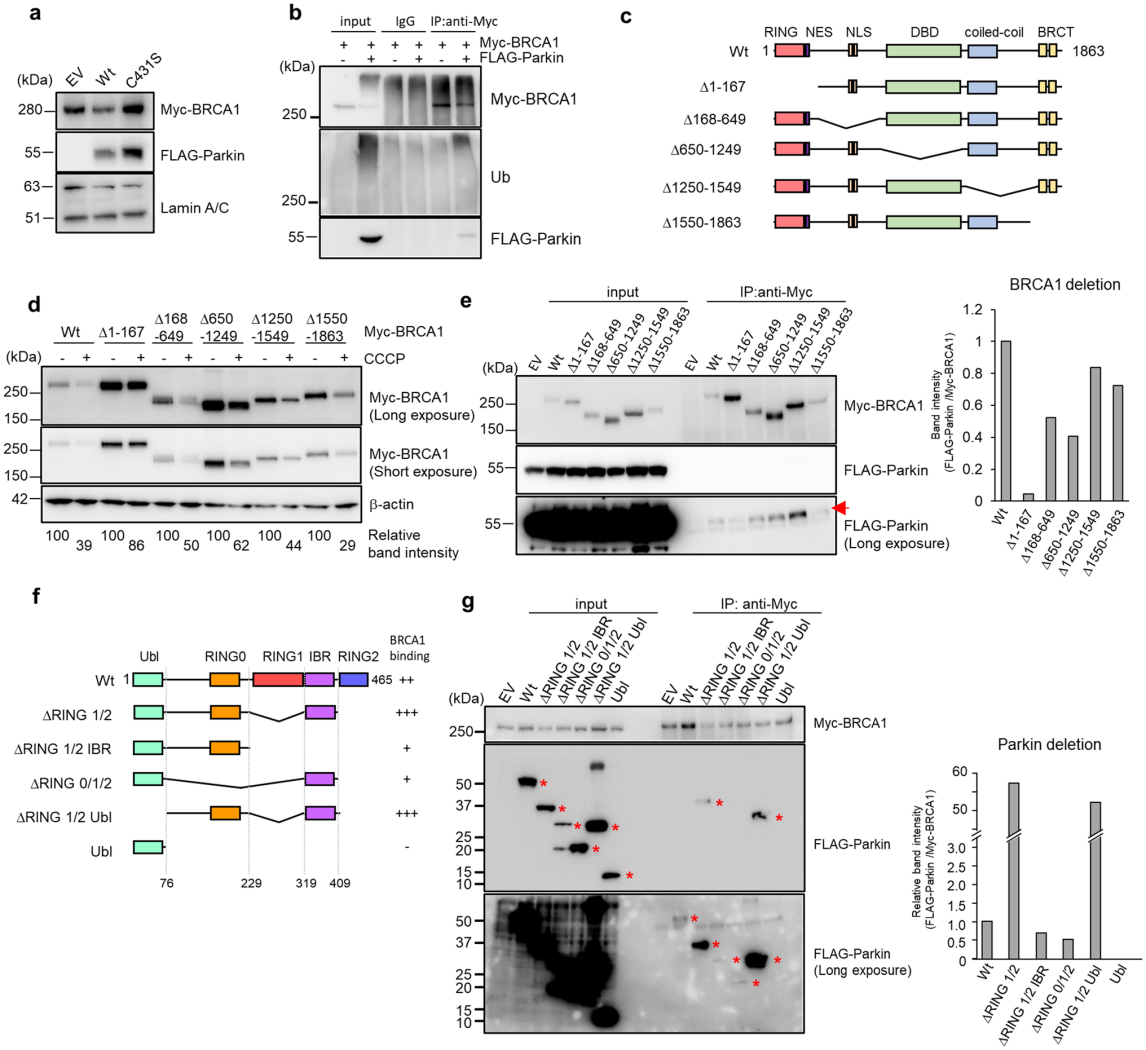
Parkin directly interacts with BRCA1. (**a**) Western blotting analysis for Myc-BRCA1 with the nuclear extract from HEK293T cells transfected with Myc-BRCA1 and empty vector (EV), wild type FLAG-Parkin (Wt), or C431S mutant FLAG-Parkin (C431S). Lamin A/C was used as a loading control. (**b**) Nuclear extracts of HEK293T cells transduced with Myc-BRCA1 and FLAG-Parkin expression vectors or an empty vector, and then treated with MG132, were subjected to Co-IP assays. Immunoprecipitates were subjected to Western blotting for Myc-BRCA1, Ub, and FLAG-Parkin. (**c**) Schematic diagram of BRCA1 expression-constructs. The degron domain is located at a.a. 1–167. The RING domain, DBD (DNA binding domain), coiled-coil domain, BRCT (BRCA1 C-terminal) domain, NES (nuclear export sequence), and NLS (nuclear localization sequence) are boxed and labeled at the top. (**d**) The expression vector for the Myc-tagged full length or deletion mutant of BRCA1 shown in (C) was transfected into HEK293T cells, which were subsequently treated with or without 10 μM CCCP for 24 h. Next, protein levels were assessed by Western blotting. Relative Myc-BRCA1 band intensity is indicated at the bottom of the figure. (**e**) Coimmunoprecipitation of FLAG-Parkin with Myc-BRCA1 deletion mutants. The arrow shows the signal of FLAG-Parkin. The band intensities of FLAG-Parkin standardized by Myc-BRCA1 in the IP samples are summarized on the right side. (**f**) Schematic diagrams of Parkin expression constructs. The binding capacity of each construct for Myc-BRCA1 is summarized on the right side. The Ubl (ubiquitin-like) domain, RING0/1/2 domains, and IBR (in-between-RING) domains are boxed and labeled at the top. (**g**) Coimmunoprecipitation of FLAG-Parkin deletion mutants with Myc-BRCA1 was analyzed by Western blotting. Asterisks indicate FLAG-Parkin. The band intensities of FLAG-Parkin standardized against Myc-BRCA1 in the IP samples are summarized on the right side.

Next, to confirm BRCA1 is directly ubiquitinated by Parkin, we coexpressed Myc-tagged BRCA1 and either wild-type or enzymatically inactive mutant (C431S) FLAG-tagged Parkin in HEK293T cells^29^. The coexpression of Myc-BRCA1 with wild-type FLAG-Parkin, but not with inactive FLAG-Parkin (C431S), decreased nuclear Myc-BRCA1 expression, indicating that BRCA1 degradation depends on the E3 ligase activity of Parkin (Fig. 3a). In immunoprecipitation (IP) experiments of nuclear extracts from coexpressing cells, ubiquitinated Myc-BRCA1 coprecipitated with FLAG-Parkin (Fig. 3b). These results suggested that Parkin interacts with and ubiquitinates BRCA1. As BRCA1 possesses a degron domain in its N-terminal region (1–167 a.a.), and this domain is ubiquitinated by several other E3 ubiquitin ligases and subsequently degraded by the ubiquitin–proteasome system^10,11,13,14^. To identify the element of BRCA1 that is responsible for CCCP-induced degradation, we constructed a series of deletion mutants of the BRCA1 protein (Fig. 3c). Following CCCP treatment, all of the Myc-BRCA1 mutants were degraded. However, BRCA1Δ1-167 was much less susceptible to degradation, suggesting that the degron domain is responsible for destruction of BRCA1 following CCCP treatment (Fig. 3d). Co-IP assays with the Myc-BRCA1 deletion mutants revealed that the N-terminal deletion (Δ1-167) indeed reduced the interaction between BRCA1 and Parkin, confirming the importance of the N-terminal degron domain of BRCA1 for direct binding with Parkin (Fig. 3e).

Conversely, we constructed deletion mutants of FLAG-Parkin to assess the domain of Parkin that is responsible for the interaction with BRCA1 (Fig. 3f). Co-IP assays revealed that Myc-BRCA1 interacted more strongly with FLAG-Parkin ΔRING1/2 than with FLAG-Parkin Wt. Because the activation of Parkin requires the dynamic rearrangement of its intramolecular domains, the deletion mutants may make the substrate recognition site more accessible^30^. Additional deletion of the Ubl domain from FLAG-Parkin ΔRING1/2 did not further weaken the interaction. However, the additional deletion of either the IBR or RING0 domain (also designated as the unique Parkin domain [UPD]) strongly inhibited the interaction. Moreover, FLAG-Ubl, generated by deletion of the IBR and RING0 domains from FLAG-Parkin ΔRING1/2, completely lost the interaction with Myc-BRCA1. These findings indicate that the IBR and RING0 domains of Parkin are required for the interaction between BRCA1 and Parkin (Fig. 3g).

### Higher BRCA1 expression correlated with lower PINK1/Parkin in breast cancer cells

**Figure 4:**
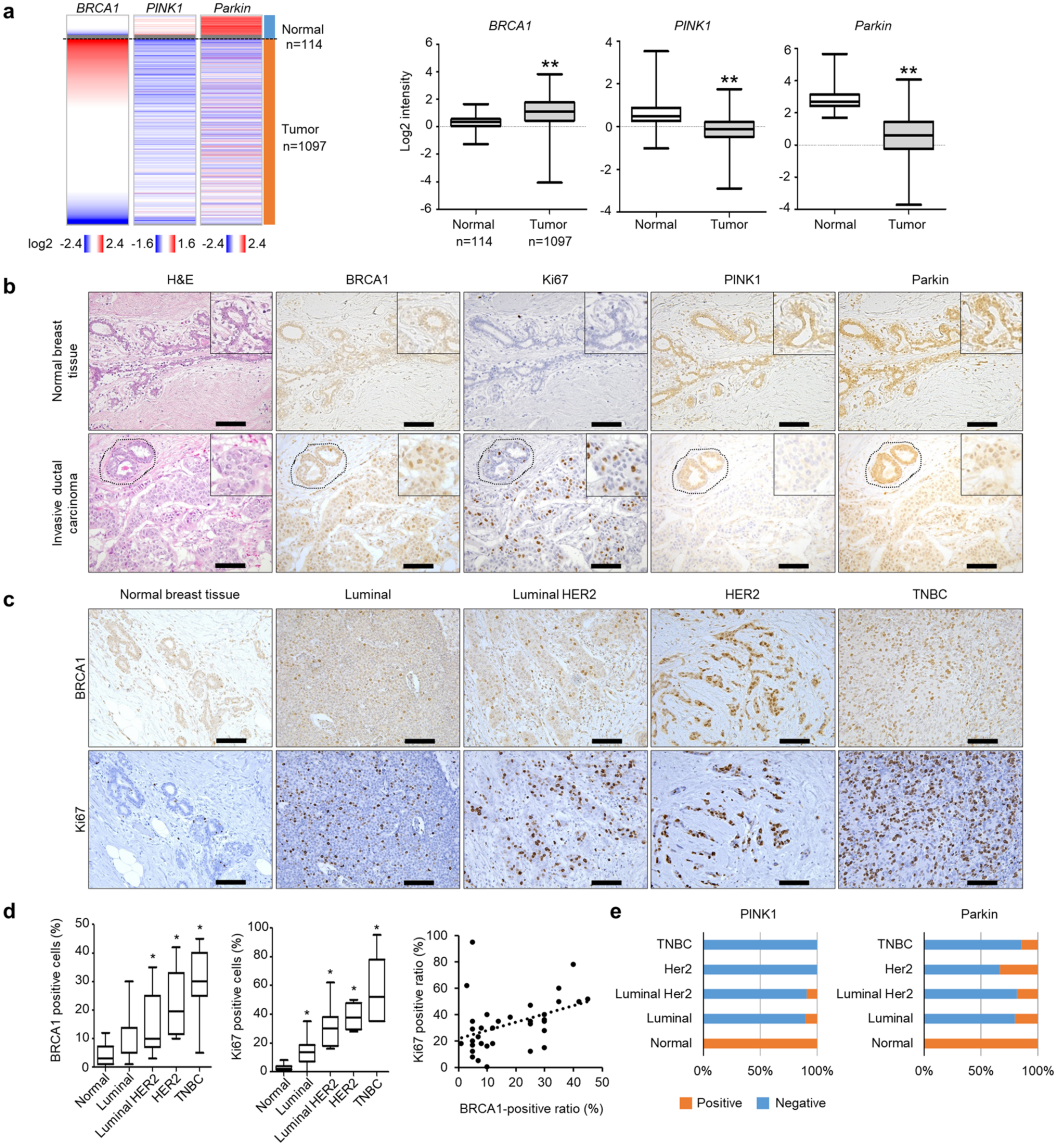
BRCA1 expression is inversely correlated with PINK1/Parkin expression in breast cancer tissues. (**a**) *BRCA1, PINK1,* and *Parkin* mRNA expression levels in human breast cancer patients were obtained from the TCGA database and summarized as a heat map (left) or box plot (right). **p < 0.01 versus normal tissues. (**b**) BRCA1, Ki67, Parkin, and PINK1 expressions in normal breast tissues and breast cancer tissues from patients were assessed by immunohistochemistry. The figures shown are representative images. On the images of invasive ductal carcinoma, circles with dashed lines indicate noncancerous breast epithelial tissues in the same images. Scale bar = 100 μm. (**c**) Immunohistochemical staining of BRCA1 and Ki67 in normal breast tissue and each subtype of breast cancer tissue. TNBC: triple negative breast cancer showing no ER, PgR, and HER2. (**d**) The left and middle graphs indicate the number of cells positive for BRCA1 or Ki67 [analyzed by IHC and shown in (C)] as a box plot. *p < 0.05 versus normal tissue. The right graph shows the correlation between the BRCA1-positive ratio and the Ki67-positive ratio in breast tumor tissue. Spearman’s correlation coefficient was analyzed (*n* = 34, *r* = 0.4245, *p* = 0.0123). (**e**) Summary of the Parkin- and PINK1-positive or -negative sample ratio in normal breast tissue and each subtype of breast cancer. Slides with Parkin- and PINK1-positive tissues were counted in each subtype, and the difference in the number of positive and negative slides between normal and tumor tissues was analyzed using the Chi-square test. The analysis revealed a significant difference in the PINK1 and Parkin expressions among the subtypes. *p* < 0.01 for Parkin and PINK1. In (**d**,**e**), the *n* for normal is 12, the *n* for luminal is 10, the *n* for luminal HER2 is 11, the *n* for HER2 is 6, and the *n* for TNBC is 7.

In the context of BRCA1 degradation via PINK1-Parkin axis shown above, we next investigated BRCA1 and PINK1/Parkin expressions in breast cancer tissues. To our surprise, analysis of publicly available data from The Cancer Genome Atlas (TCGA) revealed that breast cancer tissues express higher levels of *BRCA1*, and lower levels of *PINK1* and *Parkin* than normal breast tissues, although the *BRCA1*, *PINK1* and *Parkin* expressions did not differ significantly among the intrinsic subtypes or estrogen receptor (ER)/ progesterone receptor (PgR)/ human epidermal growth factor receptor 2 (HER2) expression profiles in breast cancers (Fig. 4a, Fig. S4). We also performed immunohistochemistry to assess BRCA1, PINK1, and Parkin expressions in breast cancer tissues derived from patients. BRCA1 expression was higher in cancerous mammary glands than in noncancerous breast epithelial tissues, whereas PINK1 and Parkin expressions were lower in tumor tissues (Fig. 4b–e). Notably, the expression of Ki67, a marker of proliferating cells, was well correlated with the expression of BRCA1 (Fig. 4c,d). These results suggest that elevated BRCA1 expression, accompanied by reduced PINK1/Parkin expression, confers a growth advantage on breast cancer cells.

### Repression of cancer cell growth upon BRCA1 downregulation

**Figure 5:**
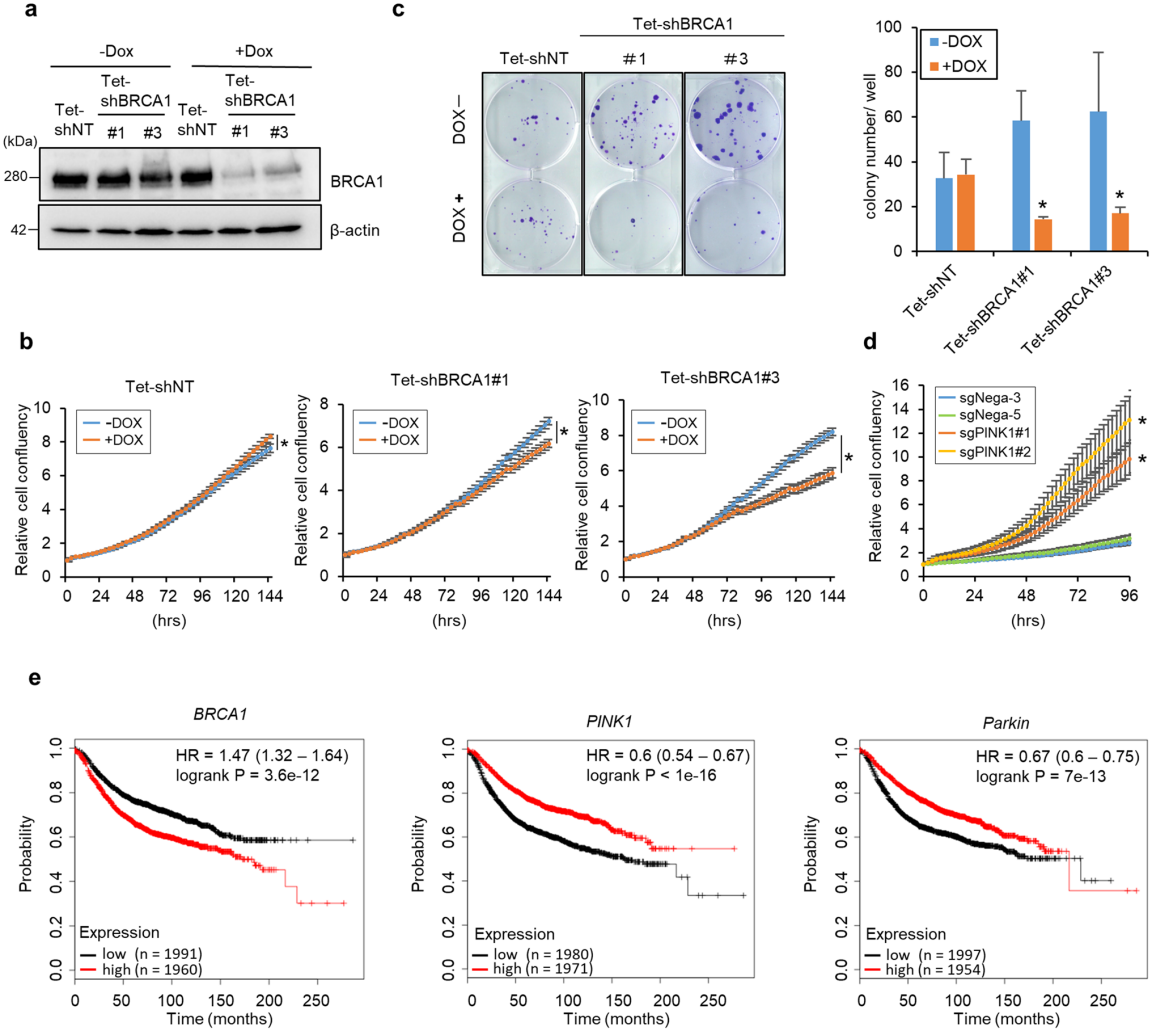
BRCA1 knockdown represses breast cancer cell growth and lower BRCA1 expression correlates with longer relapse-free survival rate. (**a**) MCF7 cells stably expressing Tet-shRNA vector against BRCA1 (Tet-shBRCA1) or control vector (Tet-shNT) were cultured with or without 0.1 μg/ml DOX for 48 h. BRCA1 knockdown efficiency was confirmed by Western blotting. (**b**) Growth of MCF7 cells expressing the Tet-shNT, Tet-shBRCA1 #1, or #3 vectors was assessed using the IncuCyte live-cell imaging system in the presence or absence of 0.1 μg/ml DOX for 4 days. *n* = 4, means ± S.D. *p < 0.05 versus -DOX. (**c**) Comparison of colony formation ability between MCF7 cells expressing Tet-shNT and Tet-shBRCA1. Cells were seeded in 6-well plates and cultured for 3 weeks in the presence or absence of 0.1 μg/ml DOX. Visualized colonies are shown in the left panel. Summarized colony numbers are shown in the right panel. Bar = means ± S.D. *n* = 3, *p < 0.05 versus -DOX. (**d**) The growth of PINK1 knockout MCF7 cells (sgPINK1#1, #2) or control cells (sgNega3, 5) was assessed using the IncuCyte live-cell imaging system. *n* = 5, means ± S.D. *p < 0.05 versus sgNega5. (**e**) Relapse-free survival rates of breast cancer patients were compared between high and low BRCA1, Parkin, or PINK1 expression in patients. Data are shown as Kaplan–Meier plots; graphs were generated using an online Kaplan–Meier plotter.

Based on the pathological findings indicating breast cancer cells express the higher BRCA1and the lower PINK1/Parkin expressions as compared with those in normal tissues (Fig. 4), we next examined the role of BRCA1 in cancer cell proliferation by knocking down BRCA1 in MCF7 cells by expressing either tetracycline-inducible BRCA1 shRNA (MCF7 Tet-shBRCA1) or nontargeting shRNA (MCF7 Tet-shNT). Knockdown efficiency following doxycycline (DOX) treatment was confirmed by Western blotting (Fig. 5a). In MCF7 Tet-shBRCA1 cells, DOX treatment significantly decreased cell growth during a 144 h-culture, whereas in MCF7 Tet-shNT cells, DOX treatment slightly increased cell growth (Fig. 5b). Clonogenic assays revealed that the colony-forming ability of MCF7 Tet-shBRCA1 cells was significantly reduced in the presence of DOX (Fig. 5c). A reduction in the number of colonies in response to BRCA1 knockdown was also observed in A549 cells (Fig. S5a,b). These data suggest that the downregulation of BRCA1 in response to mitochondrial damage attenuates cancer cell growth. Additionally, PINK1 knockout cells with an increased BRCA1 expression accelerated cell growth (Figs. 1h, 5d). This increased cell growth was dependent on increased BRCA1 expression because BRCA1 knockdown in sgPINK1#2 MCF7 cells decreased cell growth, although the cell growth was still higher than that of sgNega5 cells (Fig. S6). Moreover, PINK1 knockout in MCF7 cells changed mitochondrial morphology into elongated shapes, suggesting that PINK1-KO altered mitochondrial function and metabolism and the following cell growth (Fig. S7). In these ways, PINK1-KO may increase cell growth by BRCA1-dependent and mitochondrial function-dependent pathways. Furthermore, higher BRCA1 expression and lower PINK1/Parkin expression were significantly correlated with poor recurrence-free survival rate in breast cancer patients (Fig. 5e). These observations are particularly noteworthy given the clinical, genetic, and functional evidence that BRCA1 acts as a tumor suppressor in humans.

## Discussion

It has been well known that PINK1 is selectively stabilized on impaired mitochondria to recruit and activate Parkin, which in turn ubiquitinates MOM proteins to induce mitophagy for mitochondrial quality control^22^. We have demonstrated here a novel mechanism by which mitochondrial damage induced by mitochondria-targeting agents (e.g., CCCP) is transmitted to the nucleus in breast cancer cells. This in turn leads to nuclear BRCA1 degradation via a direct interaction with Parkin, mediated by the upregulation of PINK1 and associated with the induction of DNA double-strand breaks. Interestingly, BRCA1 knockdown attenuated cell growth and colony formation standing in stark contrast to conventional models in which BRCA1 is classified as a tumor suppressor, implying that loss of BRCA1 function would promote tumor growth (Fig. 5b,c, Fig. S5a,b)^1^. Indeed, in glioblastoma-derived cells, BRCA1 knockdown downregulates RRM2, a catalytic subunit of ribonucleotide reductase, leading to replication stress and DNA damage, and ultimately suppressing cell proliferation^31^. In the present study, we needed to use the shRNA Tet-on system for BRCA1 knockdown because the cells spontaneously recovered BRCA1 expression several passages after stable BRCA1 knockdown (data not shown), suggesting that cells with lower BRCA1 expression were negatively selected. Furthermore, BRCA1 expression correlated with the Ki67-positive ratio (Fig. 4d). Taken together, these data suggest that higher BRCA1 expression confers some growth advantage onto cancer cells, possibly reflecting the poor recurrence-free survival rate in breast cancer patients with higher BRCA1 expression (Fig. 5e). In this regard, although BRCA1 suppresses tumorigenesis, it may dichotomously promote tumor progression. This is also supported by the recent report showing that a heterodimer of BRCA1/BARD1 ubiquitinated NF2, a negative regulator of YAP1, and this leads to the stabilization of YAP1 to promote cell proliferation in the context of the Hippo pathway^32^.

In contrast to BRCA1, PINK1/Parkin was expressed at lower levels in breast cancer patients (Fig. 4). PINK1 knockout upregulated BRCA1 expression (Fig. 1h) and cell growth (Fig. 5d) in MCF7 cells. Thus, our observations indicate the tumor-suppressing role of PINK1. PINK1 knockout MCF7 cells showed a much more elongated mitochondrial morphology compared to the WT cells (Fig. S7), suggesting that PINK1 contributes to mitochondrial quality control even in the basal condition. However, a previous report showed that the downregulation of PINK1 suppressed cell growth in MCF7 cells^33^. Other reports showed that PINK1 knockdown upregulated cell growth in glioblastoma, and that ectopic overexpression of PINK1 suppressed the colony formation ability of MCF7 cells in soft agar^34,35^. Thus, the pro- and anti-tumorigenic properties of PINK1 appear to be dependent on the cellular context. Similarly to our observation, a reduction in PINK1 expression was reported in colorectal cancers^36^. In that study, the authors showed that PINK1 is negatively regulated by MYC, resulting in its suppression in tumor tissues. Another study reported that BRCA1 expression is upregulated by MYC in breast cancer cell lines^37^. Therefore, in addition to post-translational regulation of BRCA1 reported in our work here, it is possible that MYC-overexpressing cancer cells upregulate BRCA1 and downregulate PINK1 under the control of MYC, thereby protecting themselves against excess DNA double-strand breaks induced by mitochondrial damage and maintaining cell growth.

In addition to MOM proteins, the substrates for Parkin include nuclear proteins and transcriptional factors, including hypoxia-inducible factor 1α(HIF-1α)^29,38,39^. HIF-1α transcriptionally regulates gene expression and promotes tumor malignancy^40^. Because Parkin negatively regulates HIF-1α, the expression levels of Parkin and HIF-1α are inversely correlated in human breast cancer tissues, and lower Parkin expression is correlated with poor metastasis-free survival in breast cancer patients^38^; as shown in the present study, a similar relationship holds for Parkin and BRCA1 (Fig. 4). In this regard, Parkin appears to function as a tumor suppressor, as previously reported^41^. However, the major driving force underlying the induction of HIF-1α ubiquitination by Parkin remains unclear. We found that mitochondrial damage induces a direct interaction between BRCA1 and ectopically expressed Parkin in HEK293T cells (Fig. 3). However, owing to the low expression of Parkin in cancer cells, we could not conclusively determine whether the knockdown of endogenous Parkin attenuates CCCP-induced BRCA1 degradation in MCF7 and A549 cells (data not shown). Given that the knockout of endogenous PINK1 resulted in the upregulation of BRCA1 as well as the attenuation of CCCP-induced BRCA1 degradation in MCF7 cells (Fig. 1h), it remains possible that E3 ligases other than Parkin activated by PINK1 might also be involved in BRCA1 ubiquitination upon mitochondrial damage.

Emerging evidence has implicated DNA damage in the pathogenesis of neurodegenerative diseases^42^. Specifically, BRCA1 expression is reduced in the brains of patients with Alzheimer’s disease (AD), as well as in the brain of an AD mouse model expressing human amyloid-β precursor^43^. On the other hand, Mano et al*.* reported that the *BRCA1* promoter is hypomethylated, and BRCA1 expression is elevated in AD brains. However, they also reported that BRCA1 was mislocalized to the cytoplasm as highly insoluble aggregates in a tau-dependent manner^44^. Both reports showed correlations between AD and BRCA1 dysfunction, accompanied by DNA instability. In the present study, we also observed that BRCA1 was degraded by mitochondrial damage in neuroblastoma-derived SH-SY5Y cells (Fig. 1a). Thus, the transmission of mitochondrial damage to the nucleus, where it manifests as DNA double-strand breaks via the PINK1–Parkin–BRCA1 axis, may be relevant not only in the field of oncology, but also in the study of neurodegenerative diseases.

## Materials and methods

### Human specimens

Thirty-four pairs of primary tumor and adjacent nonmalignant surgical tissue specimens were obtained from patients with breast cancer who underwent surgery before receiving neoadjuvant chemotherapy at Tokyo Medical University Hospital from 2016 to 2017. The patients’ background information and clinicopathological characteristics are presented in Table S1. This study was approved by the Institutional Review Board (IRB) of Tokyo Medical University (approval ID: T2018-0022) and was performed in accordance with the regulations and guidelines set by the IRB. In accordance with the IRB, we did not obtain the written forms of informed consent from the patients regarding the use of their clinical data for this study. Tokyo Medical University Hospital obtains the comprehensive agreement from patients at their first consultation about the usage of tissue specimen derived from patients for a variety of medical research, and information about this study was disclosed to the patients on the hospital homepage and outpatient bulletin board to ensure that the patients can refuse to be enrolled in the study. The data from patients were analyzed anonymously.

### Cell culture

All cell lines used in this study were obtained from ATCC (Manassas, VA, USA). MCF7, A549, and SH-SY5Y cells were cultured in Dulbecco’s Modified Eagle’s medium (DMEM), and SKBR3 cells were cultured in McCoy’s5A supplemented with 10% FBS and penicillin/streptomycin in a humidified 5% CO_2_ incubator at 37 °C. MDA-MB-231 and MDA-MB-468 cells were cultured in Leibovitz’s L-15 medium supplemented with 10% FBS and penicillin/streptomycin in a humidified incubator at 37 °C. For mitochondrial inhibition, carbonyl cyanide m-chlorophenyl hydrazine (CCCP) (Sigma-Aldrich; Merck KGaA, Darmstadt, Germany) treatment was performed at 2.5, 5, or 10 μM for an optimal hour. MG132 (Peptide Institute, Osaka, Japan) and N-acetyl cysteine (NAC) (Nacalai Tesque, Kyoto, Japan) treatments were performed at 10 μM and 10 mM, respectively, 30 min before CCCP treatment. For alternative mitochondrial inhibition, cells were treated with antimycin A (Santa Cruz Biotechnology, Dallas, TX, USA), oligomycin A (Cayman Chemicals, Ann Arbor, MI, USA), deferiprone (DFP) (Tokyo Chemical Industry, Tokyo, Japan), rotenone (Cayman), or valinomycin (Cayman) for 24 h. Cells were cultured up to 1 month after thawing. Mycoplasma contamination was routinely tested using the e-Myco Mycoplasma PCR Detection kit (iNtRON Biotechnology, Inc., Korea).

### Western blotting

To obtain whole-cell lysates, cells were harvested and lysed with 1 × RIPA buffer (Nacalai Tesque) containing protease inhibitor cocktail (Nacalai Tesque) and phosphatase inhibitor cocktail (Nacalai Tesque). Cell lysates were separated by sodium dodecyl sulfate polyacrylamide gel electrophoresis (SDS-PAGE) and blotted on a polyvinylidene fluoride membrane. Western blotting was performed with the following antibodies: anti-BRCA1 (Cell Signaling Technology [CST], Danvers, MA, USA #9010), anti-β-actin (Santa Cruz Biotechnology #47778), anti-TOM20 (Santa Cruz Biotechnology #11415), anti-COX IV (CST #11967), anti-FLAG (SIGMA #F1804), anti-γH2AX (Millipore Merck #05-636), anti-PINK1 (CST #6946), anti-Myc (CST #2276), and anti-Parkin (Santa Cruz Biotechnology #32282). Intensity of each protein band was quantified using ImageJ software (https://imagej.nih.gov/ij/). Each band intensity was standardized by β-actin. Uncropped original images merged with corresponding marker images are shown in Fig. S8-17.

### Vector construction

Myc-tagged BRCA1 expression vectors used for co-IP assay were constructed with pcDNA6/V5-His expression vector (Thermo Fisher Scientific, Waltham, MA, USA) without any tags at the C-terminal. FLAG-Parkin expression vectors used for co-IP assay were constructed with p3xFLAG-CMV-10 expression vector (SIGMA). Knockout vectors for PINK1 were constructed with pSpCas9(BB)-2A-Puro (PX459) V2.0 vector (gift from Dr. Feng Zhang: Addgene # 62988)^45^ using the sequence shown in Table S2.

### Lentiviral introduction

Lentiviruses were produced in HEK293T cells by transfection of the following plasmids: pMD2.G (gift from Didier Trono: Addgene#12259), psPAX2 (gift from Dr. Didier Trono: Addgene#12260), and pLKO.1 shRNA expression vector (gift from Dr. Bob Weinberg: Addgene #8453)^46^, Tet-pLKO-puro shRNA expression vector (gift from Dr. Dmitri Wiederschain: Addgene#21915)^47^, or N-terminal 3xFLAG tagged Parkin inserted pLentiN vector (gift from Dr. Karl Munger: Addgene #37444)^48^. shRNA vectors targeting BRCA1, Parkin, and PINK1, and an shNT non-targeting control vector were constructed using the sequence shown in Table S2.

### Establishment of knockout cell lines

To establish the knockout cells, the CRISPR-Cas9 system was used. MCF7 cells were transfected with pCas9-PINK1#1 or pCas9-PINK1#2 vectors (Table S2) to knockout PINK1 or transfected with pCas9-Nega to establish negative control clones. Thereafter, cells were selected with puromycin for 2 days and grown without puromycin to pick up single colonies.

### Immunofluorescence

For immunofluorescence staining of γH2AX, cells were fixed with a methanol and acetone mixture at − 20 °C. For immunofluorescence staining of BRCA1 and FLAG-Parkin, cells were fixed with 2% paraformaldehyde for 10 min. After blocking with 10% normal goat serum in TBST, cells were incubated with primary antibody (anti-γH2AX CST #9718; 1:100, anti-BRCA1 CST #9010; 1:200, anti-FLAG-M2 Sigma; 1:1000) diluted in blocking reagent for 16 h at 4 °C. Then, primary antibodies were detected with anti-mouse IgG and/or anti-rabbit IgG conjugated to Alexa Fluor 488 or Alexa Fluor 555 antibodies (Invitrogen, Waltham, MA, USA). Nuclei were stained with 4′,6-diamidino-2-phenylindole (DAPI). Fluorescence signals were observed by confocal microscopy (LSM7000, Carl Zeiss, Germany). All images in each figure were acquired and processed in the same manner using ZEN 2012 software (Carl Zeiss).

### Cell fractionation

For fractionation into the cytoplasm, nucleus, and mitochondria, cells were separated using the WSE-7422 EzSubcell Fraction kit (ATTO, Tokyo, Japan). Each separated fraction was used for Western blotting.

### Immunoprecipitation

Immunoprecipitation was carried out on whole-cell lysates or nuclear extracts. Whole-cell lysates were prepared in 1 × Lysis buffer (CST) containing a protease inhibitor cocktail and a phosphatase inhibitor cocktail. To prepare nuclear extracts, cells were homogenized using a Dounce homogenizer in hypotonic buffer (10 mM HEPES pH 7.9, 1.5 mM MgCl_2_, 10 mM KCl, protease inhibitor cocktail, and phosphatase inhibitor cocktail), and centrifuged for 15 min at 3,300 g. Thereafter, precipitations were suspended in hypotonic buffer to which equal volumes of high-salt buffer (20 mM HEPES pH 7.9, 25% glycerol, 1.5 mM MgCl_2_, 0.8 M KCl, 0.2 mM EDTA, and 1 mM DTT) were added, and then rotated for 30 min at 4 °C. Then, the lysates were centrifuged at 20,000*g* for 30 min, and the supernatant was used as nuclear extract. For immunoprecipitation, lysates were incubated with anti-Myc antibody (CST) for 16 h, and then incubated with SureBeads Protein G Magnetic Beads (Bio-Rad, Hercules, CA, USA). Beads were washed with 1 × lysis buffer (CST), and then eluted with 2 × SDS-PAGE sample buffer.

### Colony formation assay

MCF7 Tet-shNT or Tet-shBRCA1 cells were seeded in 6-well plates at 500 cells per well, and then cultured for 3 weeks with or without 0.1 μg/mL doxycycline. After culture, the cells were fixed with 10% neutral formalin and stained with crystal violet^49^. Colonies were counted using the IncuCyte Zoom imaging system (Essen BioScience, Ann Arbor, MI, USA). A549 Tet-shNT or Tet-shBRCA1 cells were seeded in 6-well plates at 100 cells per well, and cultured for 11 days with or without 0.1 μg/mL doxycycline.

### Cell proliferation assay

MCF7 Tet-shNT and MCF7 Tet-shBRCA1 #1, #3 cells were seeded at 1 × 10^4^ cells/well in 48-well plates 16 h before the assay to allow them to adhere to the plate bottom. Then, the cells were cultured with or without 0.1 μg/mL doxycycline for 6 days, and cell confluency was monitored every 2 h using the IncuCyte Zoom live-cell imaging system (Essen BioScience).

### Database analysis

To analyze BRCA1, PINK1, and Parkin expressions in the publicly available dataset “TCGA breast invasive carcinoma (BRCA) gene expression by RNA-seq” (dataset ID: TCGA.BRCA.sampleMap/HiSeqV2), we used the UCSC Xena platform^50^. Relapse-free survival data of breast cancer patients were obtained using an online Kaplan–Meier plotter^51^. To stratify patients into high- and low-expression groups, the median value of each gene’s expression was used.

### Measurement of mitochondrial membrane potential

Mitochondrial membrane potential was assessed with tetramethylrhodamine, ethyl ester (TMRE) (Thermo). Cells were treated with a vehicle, CCCP, OA, valinomycin, or DFP for 24 h. After treatment, a TMRE probe was added to each cell. The cells were then incubated in a CO_2_ incubator for 30 min, washed twice with PBS, and detached from the dish. TMRE fluorescence was measured using an Attune flow cytometer (Thermo).

### Immunohistochemical analysis of human breast cancer patients’ specimens

Immunohistochemical analysis of breast cancer tissues was performed with anti-PINK1 (BC100-494, Novus Biologicals, Centennial, CO, USA), anti-Parkin (sc-32282, Santa Cruz Biotechnology), anti-BRCA1 (MS110, Abcam, Cambridge, UK), and anti-Ki67 (MIB-1, M7240, DAKO, CA, USA) antibodies. Paraffin sections were deparaffinized and rehydrated. Antigen retrieval was performed by autoclaving at 105ºC for 20 min with a retrieval solution pH 9.0 (Nichirei Biosciences Inc., Tokyo) for BRCA1, PINK1 and Parkin, or by autoclaving at 121ºC for 10 min with 1 mM Tris/EDTA pH 9.0 for Ki67. For these analyses, tissues showing atypical ductal hyperplasia were excluded. To quantify BRCA1-positive cells, only nuclear signals were used. For the scoring of PINK1 and Parkin expressions in breast cancer tissues, we compared the signal intensity with normal tissues; a signal was counted as negative only when the signal intensity was lower than the intensity in the normal tissues.

### Real-time PCR

Total RNA was isolated using NucleoSpin RNA (Machery-Nagel GmbH, Düren, Germany) and treated with DNase I; cDNA was synthesized using PrimeScript RT Master Mix (Takara). Real-time PCR was performed using SYBR Premix Ex Taq II (Takara) with primers listed in Table S2. Target mRNA expression was calculated based on the ΔΔCt method. *HPRT1* mRNA was used as an internal control.

### Quantification and statistical analysis

Differences in the growth of MCF7 cells expressing Tet-shNT or Tet-shBRCA1 #1 and #3 were analyzed by two-way ANOVA using GraphPad Prism. Spearman’s correlation coefficient between the BRCA1-positive ratio and Ki67-positive ratio in breast cancer tissues was also analyzed using GraphPad Prism. All other differences were analyzed using one-way ANOVA, followed by Dunnett’s multiple comparison test or Tukey’s multiple comparison test. A *p-*value < 0.05 was considered to indicate a statistically significant difference.

## Supplementary Information

Supplementary Information
