# Supplementary material for: BRCA1 degradation in response to mitochondrial damage in breast cancer cells
Source: Sci Rep. 2021 Apr 22;11:8735. doi: 10.1038/s41598-021-87698-7 (PMC8062582; doi:10.1038/s41598-021-87698-7)
Supplement: Supplementary file 1 — Supplementary Information [file 41598_2021_87698_MOESM1_ESM.pdf]

Supplementary information

BRCA1 degradation in response to mitochondrial damage in breast cancer cells

Kana Miyahara[a,1], Naoharu Takano[b,1], Yumiko Yamada[b], Hiromi Kazama[b], Mayumi Tokuhisa[b], Hirotsugu Hino[b], Koji Fujita[c], Edward Barroga[d], Masaki Hiramoto[b], Hiroshi Handa[e], Masahiko Kuroda[c], Takashi Ishikawa[a], Keisuke Miyazawa[b]

[a]Department of Breast Oncology and Surgery, [b]Department of Biochemistry, [c]Department of Molecular Pathology, and [e]Department of Nanoparticle Translational Research, Tokyo Medical University, Shinjuku, Tokyo 160-8402, Japan; [d]St. Luke's International University, Tokyo 104-0044, Japan. [1]These authors contributed equally.

Supporting Information

Materials and Methods

Cell lines

ATG5-KO A549 cells were established as shown previously (hino et. al.). Control and PINK1 KO A549 cells were established with pCas9-sgNega and pCas9-sgPINK1#1 vectors. MCF7 sgNega5-Tet-shNT, sgNega-Tet-shBRCA1#3, sgPINK1-Tet-shNT, and sgPINK1#2-Tet-shBRCA1#3 were established by lentivirus infection and selection with puromycin. PINK KO MDA-MB-231 and MDA-MB-468 cells were established as shown in "Materials and Methods" in main text.

Cell cycle analysis

Cells were cultured in the presence or absence of the 10 μM CCCP for 24 hours, and then harvested with trypsin. Nuclear DNA was stained with CyStain DNA 2 step kit (Sysmex, Kobe, Japan) and measured by flow cytometer. The cell cycle profile was analyzed with ModFit LT (Verity Software House, Topsham, ME, USA).

References

1. Hino H, et al. Abemaciclib induces atypical cell death in cancer cells characterized by formation of cytoplasmic vacuoles derived from lysosomes. Cancer Sci 111, 2132-2145 (2020).

Patient #	Invasive tumor size (cm)	Node number	Pathological Stage	Subtype
1	1.5	0	I	Luminal
2	4.4	6	IIIA	Luminal
3	1.5	0	I	Luminal
4	2.0	0	I	Luminal
5	2.0	0	I	Luminal
6	1.8	0	I	Luminal
7	3.8	0	II	Luminal
8	6.2	6	IIIB	Luminal
9	3.5	0	II	Luminal
10	1.6	1	IIA	Luminal
11	1.6	1	IIA	Luminal HER2
12	2.7	2	Ⅱ B	Luminal HER2
13	1.2	0	I	Luminal HER2
14	1.6	2	IIA	Luminal HER2
15	1.6	0	I	Luminal HER2
16	1.9	0	I	Luminal HER2
17	1.5	0	I	Luminal HER2
18	1.7	1	IIA	Luminal HER2
19	2.2	1	IIB	Luminal HER2
20	3.2	0	IIA	Luminal HER2
21	1.3	1	IIA	Luminal HER2
22	0.6	0	I	HER2
23	0.7	0	I	HER2
24	0.8	0	I	HER2
25	0.5	0	I	HER2
26	2.5	0	IIA	HER2
27	0.5	1	IIA	HER2
28	1.2	0	I	Triple-negative
29	9.0	-	-	Triple-negative
30	0.7	0	I	Triple-negative
31	2.5	0	IIA	Triple-negative
32	4.4	4	IIIA	Triple-negative
33	1.3	0	I	Triple-negative
34	1.8	0	I	Triple-negative

Table S1. Clinicopathological characteristics of breast cancer patients. Related to Methods.

Designation	Sequence
**DNA sequence for shRNA vector**	
shNT	5’-CCGGCAACAAGATGAAGAGCACCAACTCGAGTTGGTGCTCTTCATCTTGTTGTTTTTG-3’
shBRCA1#1	5’-CCGGGAGTATGCAAACAGCTATAATCTCGAGATTATAGCTGTTTGCATACTCTTTTTTG-3’
shBRCA1#3	5’-CCGGACTGATACTGCTGGGTATAATCTCGAGATTATACCCAGCAGTATCAGTTTTTTTG-3’
**DNA sequence for pCas9 vector**	
sgNega	5’-CACCGGTAGCGAACGTGTCCGGCGT-3’
sgPINK1#1	5’-CACCGCGTCTCGTGTCCAACGGGTC-3’
sgPINK1#2	5’-CACCGCGCCACCATGGCGGTGCGAC-3’
**DNA sequence for real-time PCR**	
hBRCA1 Fwd	5’-GAAACCGTGCCAAAAGACTTC-3’
hBRCA1 Rev	5’-CCAAGGTTAGAGAGTTGGACAC-3’
hHPRT1 Fwd	5’-GAAAAGGACCCCACGAAGTGT-3’
hHPRT1 Rev	5’-AGTCAAGGGCATATCCTACAACA-3’

Table S2. shRNA and primer sequences. Related to Methods. The DNA sequences used for shRNA vector and pCas9 vector construction and the primer sequences used for real-time PCR are listed.

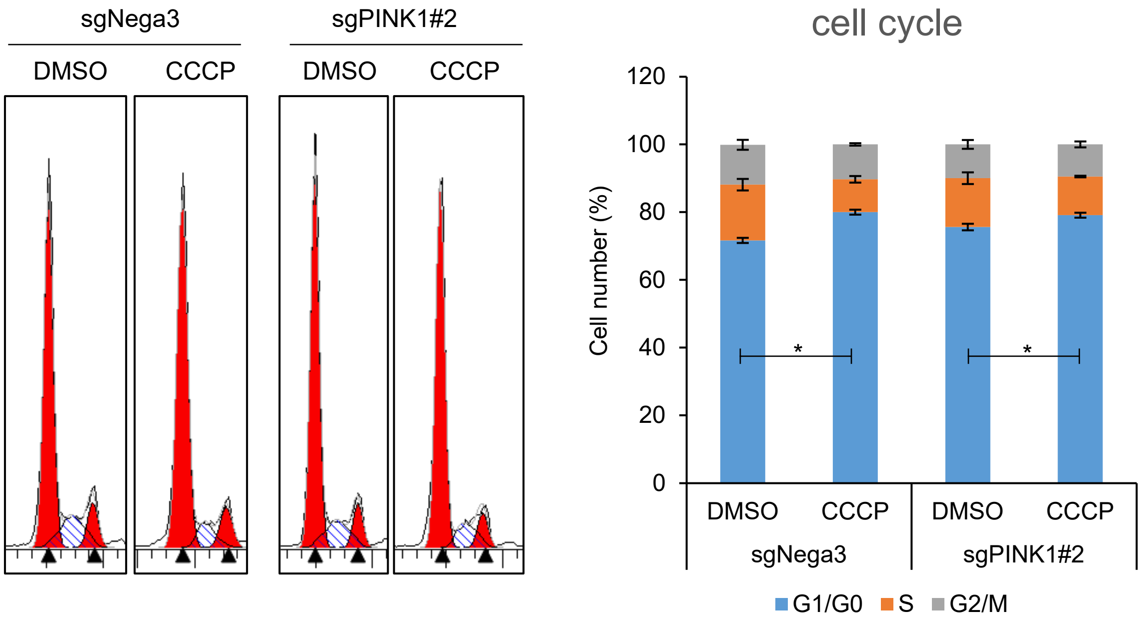

Fig. S1. G0/G1 phase was increased by CCCP treatment in control cells as well as PINK1 knockout cells. Cell cycle profiles were analyzed by flow cytometer. Representative data of DNA contents distribution was shown in left and summarized graph was shown in right. Bar = means ± S.D., n = 5, *p < 0.05 versus DMSO.

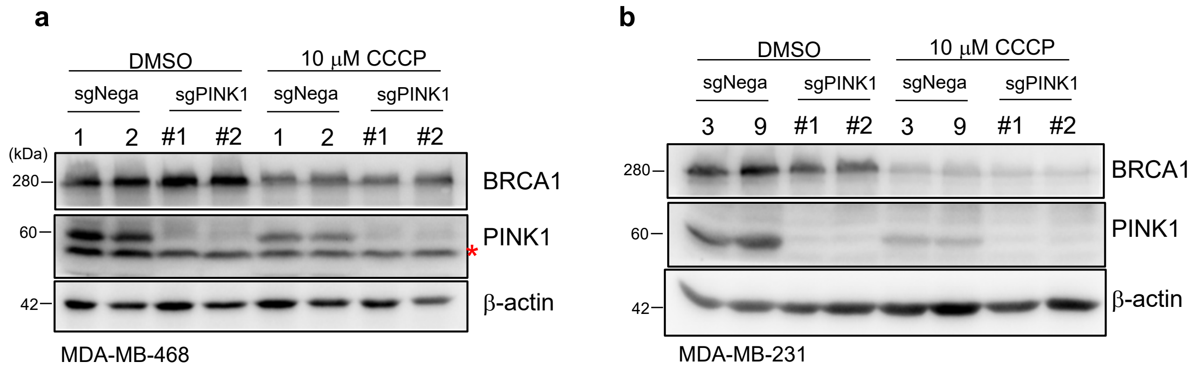

Fig. S2. PINK1-KO in MDA-MB-468 and MDA-MB-231 cells. (**a**) Control or PINK1-KO MDA-MB-468 cells were treated with CCCP for 24 h and the BRCA1 and PINK1 expression levels were assessed. The asterisk indicates a non-specific band. (**b**) Control or PINK1-KO MDA-MB-231 cells were treated with CCCP for 24 h and assessed for BRCA1 and PINK1 expression levels.

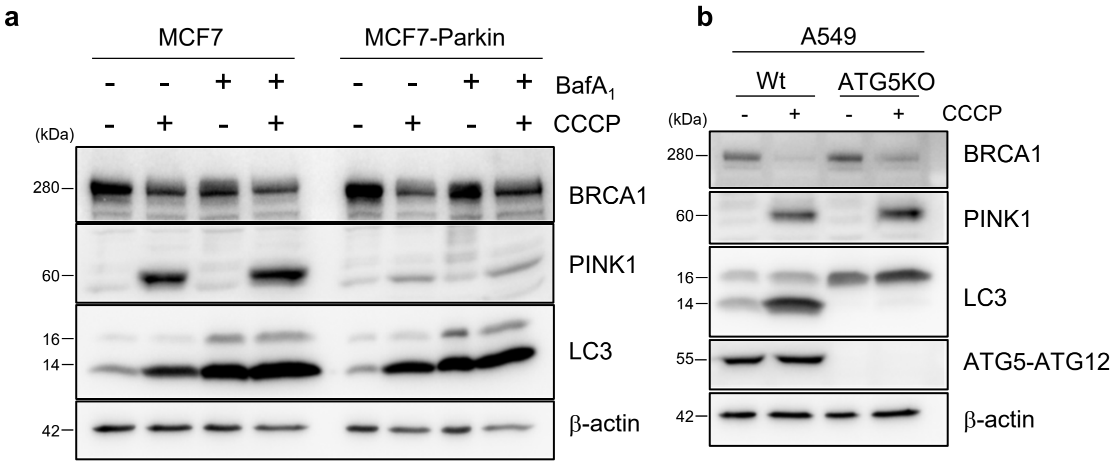

Fig. S3. BRCA1 degradation is independent from autophagy. (**a**) MCF7 cells and FLAG-Parkin expressing MCF7 cells were treated with 10 μM CCCP and 10 nM bafilomycin A_1 for 24 h, and then BRCA1 protein level was assessed by Western blotting. (**b**) Wild type and ATG5-KO A549 cells were treated with CCCP for 24 h.

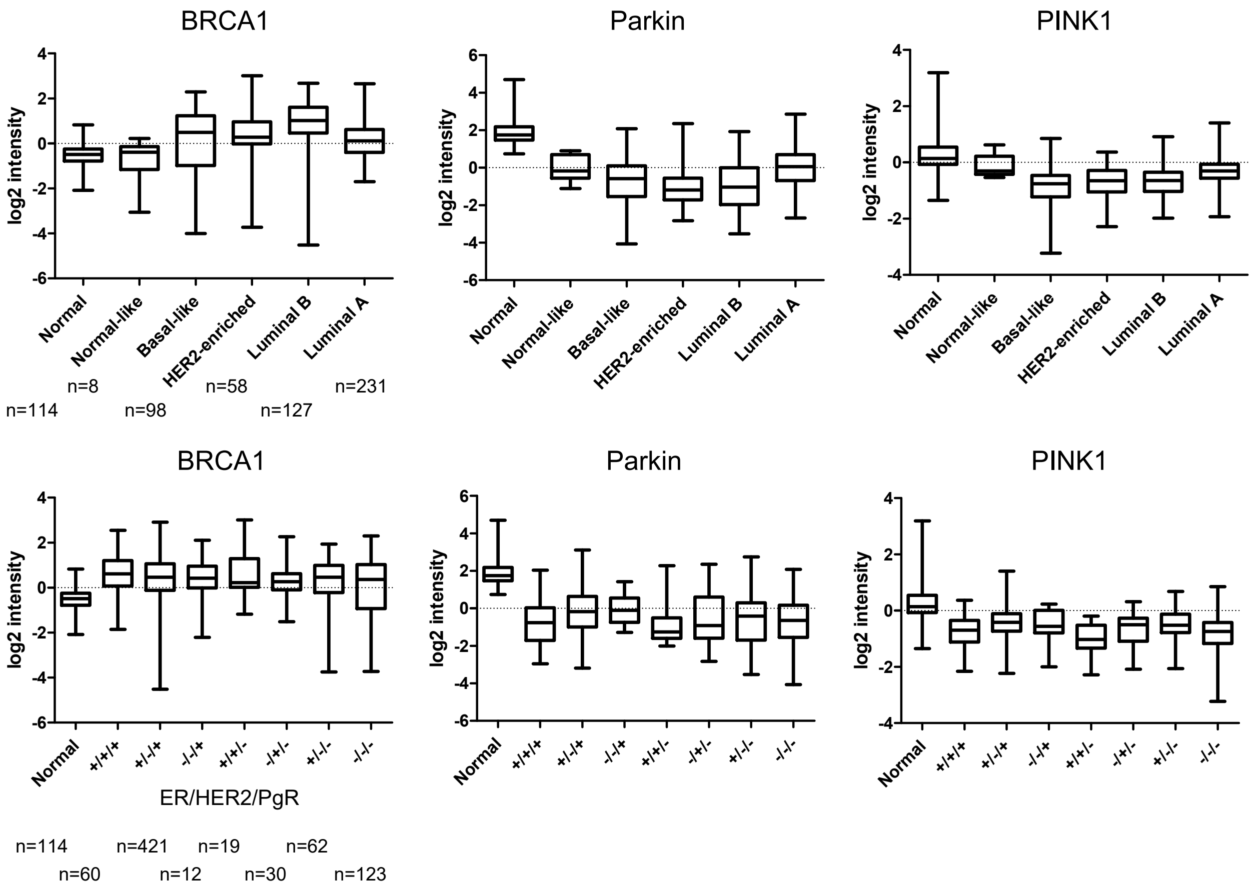

Fig. S4. BRCA1, Parkin, and PINK1 expressions in different breast cancer subtypes. Gene expression profiles of BRCA1, Parkin, and PINK1 were obtained from the publicly available TCGA database. Breast cancer samples were classified depending on the intrinsic subtypes or ER/PR/HER2 expression pattern, and then compared.

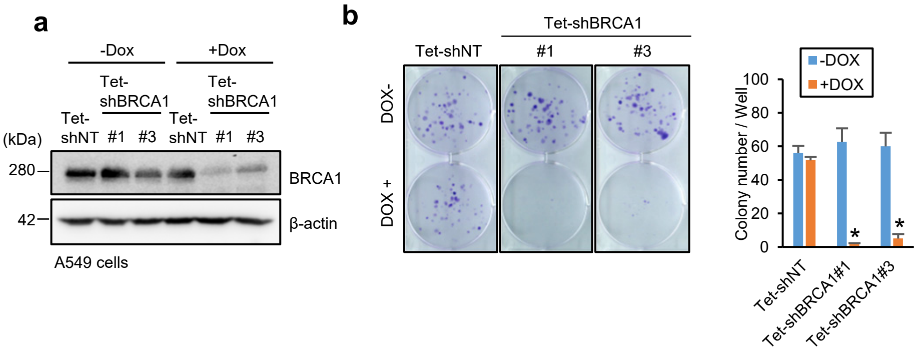

Fig. S5. BRCA1 knockdown suppressed the colony formation ability of A549 cells. (**a**) Tetracycline-inducible shRNA against BRCA1 or control vector was stably introduced into A549 cells. BRCA1 knockdown efficiency was assessed by Western blotting after 0.1 μg/mL DOX treatment for 2 days. (**b**) Colony formation ability in the presence or absence of DOX. Visualized colonies are shown on the left side, and colony numbers (i.e., graph) are shown on the right side. Bar = means ± S.E., $n = 3$, *$p < 0.05$ versus -DOX.

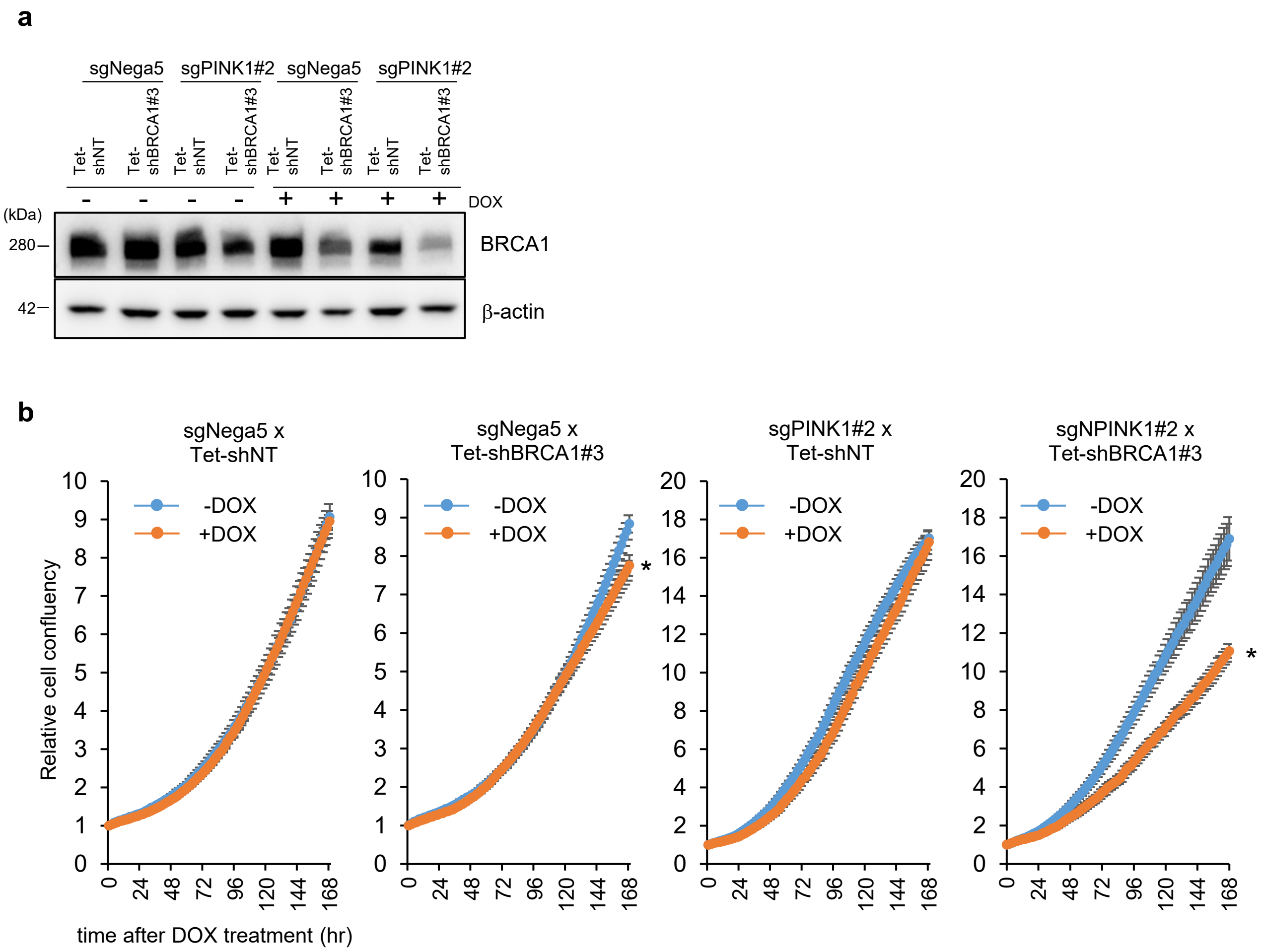

Fig. S6. BRCA1 knockdown in PINK1-KO MCF7 cells reduced cell growth. (**a**) sgNega5 and sgPINK1#2 MCF7 cells were introduced with Tet-shNT or Tet-shBRCA1#3 knockdown vector. BRCA1 expression was assessed after 0.1 μg/mL DOX treatment for 48 h. (**b**) The cell growth of each type of cells was analyzed using the IncuCyte live-cell imaging system in the presence or absence of DOX (0.1 μg/ml). Bar = mean ± S.E., n = 4, *$p < 0.05$ versus -DOX.

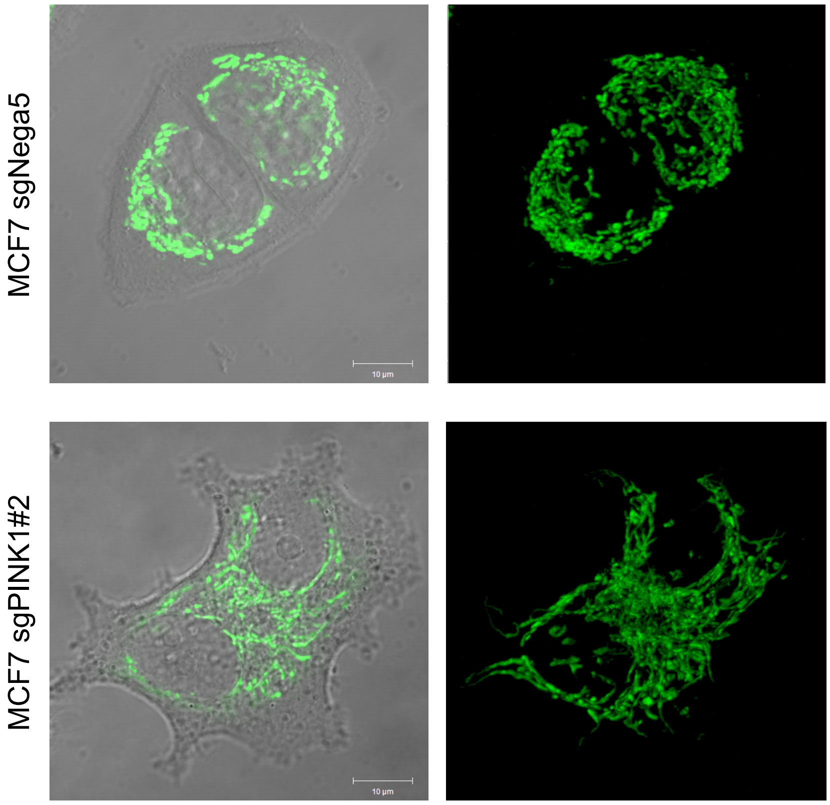

Fig. S7. Mitochondrial morphology in PINK1-KO cells. Mitochondria were stained with Mito Tracker Green FM (Green) and observed by confocal microscopy. The right panels show z-stacked images. Mitochondria in PINK1-KO MCF7 cells exhibited an elongated morphology. Scale bar = 10 µm.

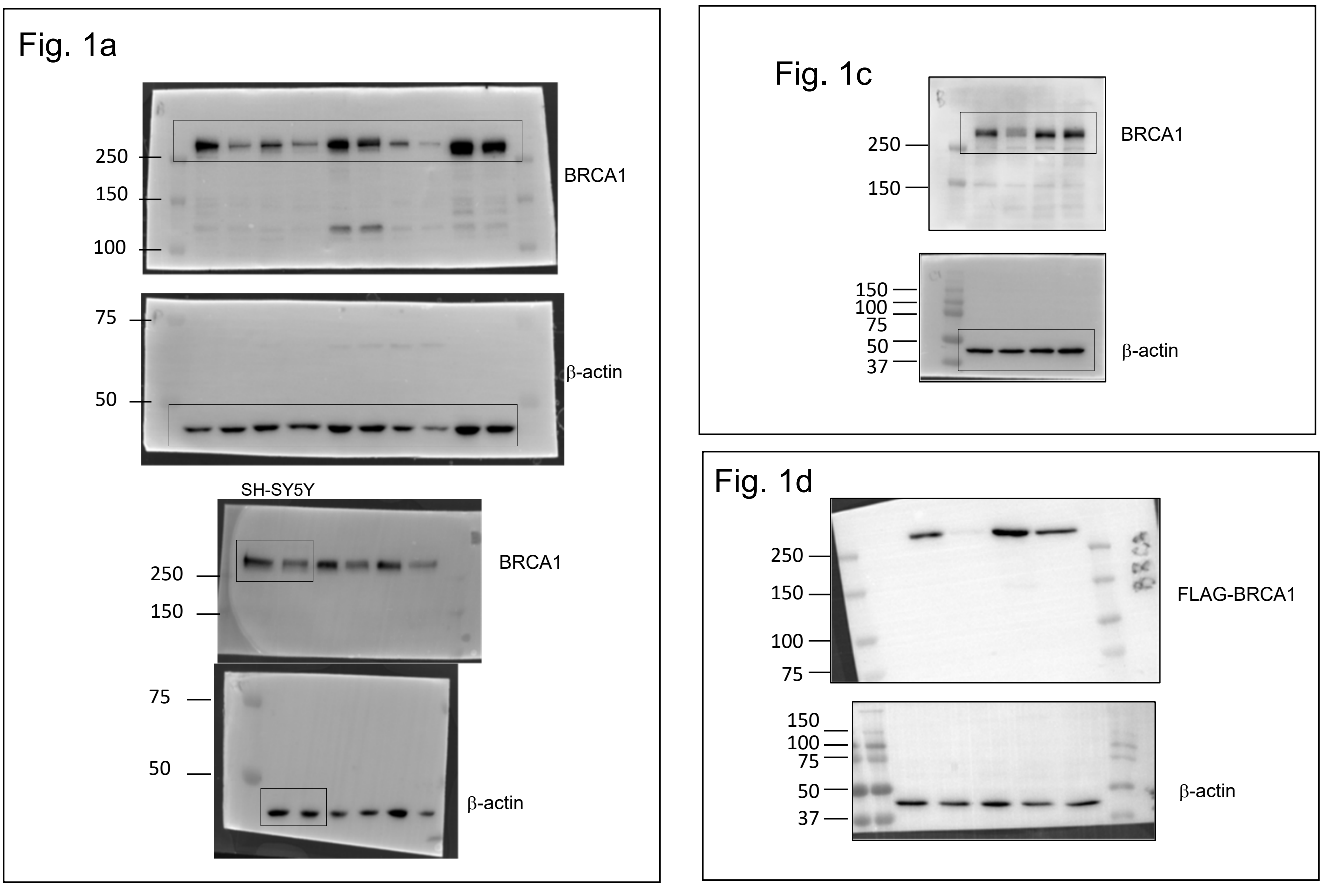

Figure S8. Original immunoblot images merged with corresponding marker images.

Fig. 1e

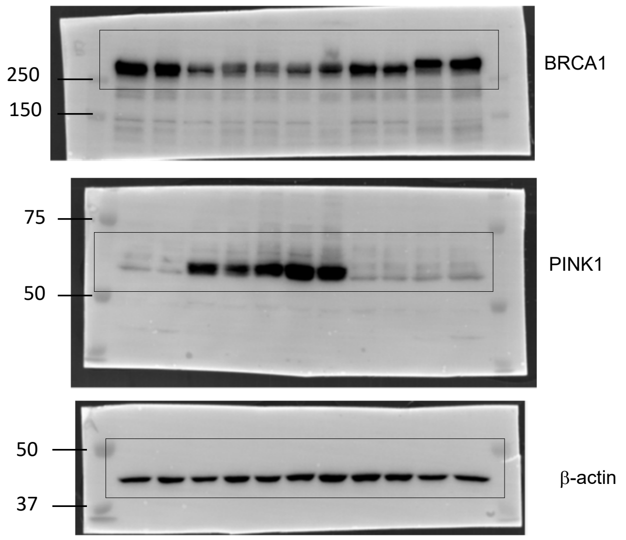

Fig. 1f (Source Data used for statistical analysis)

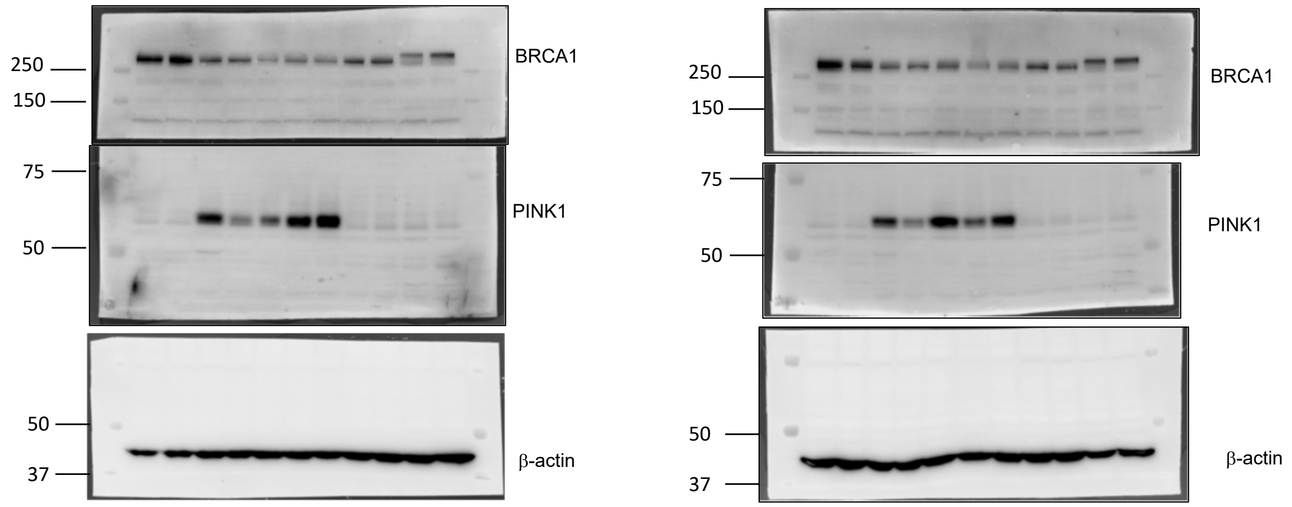

Figure S9. Original immunoblot images merged with corresponding marker images.

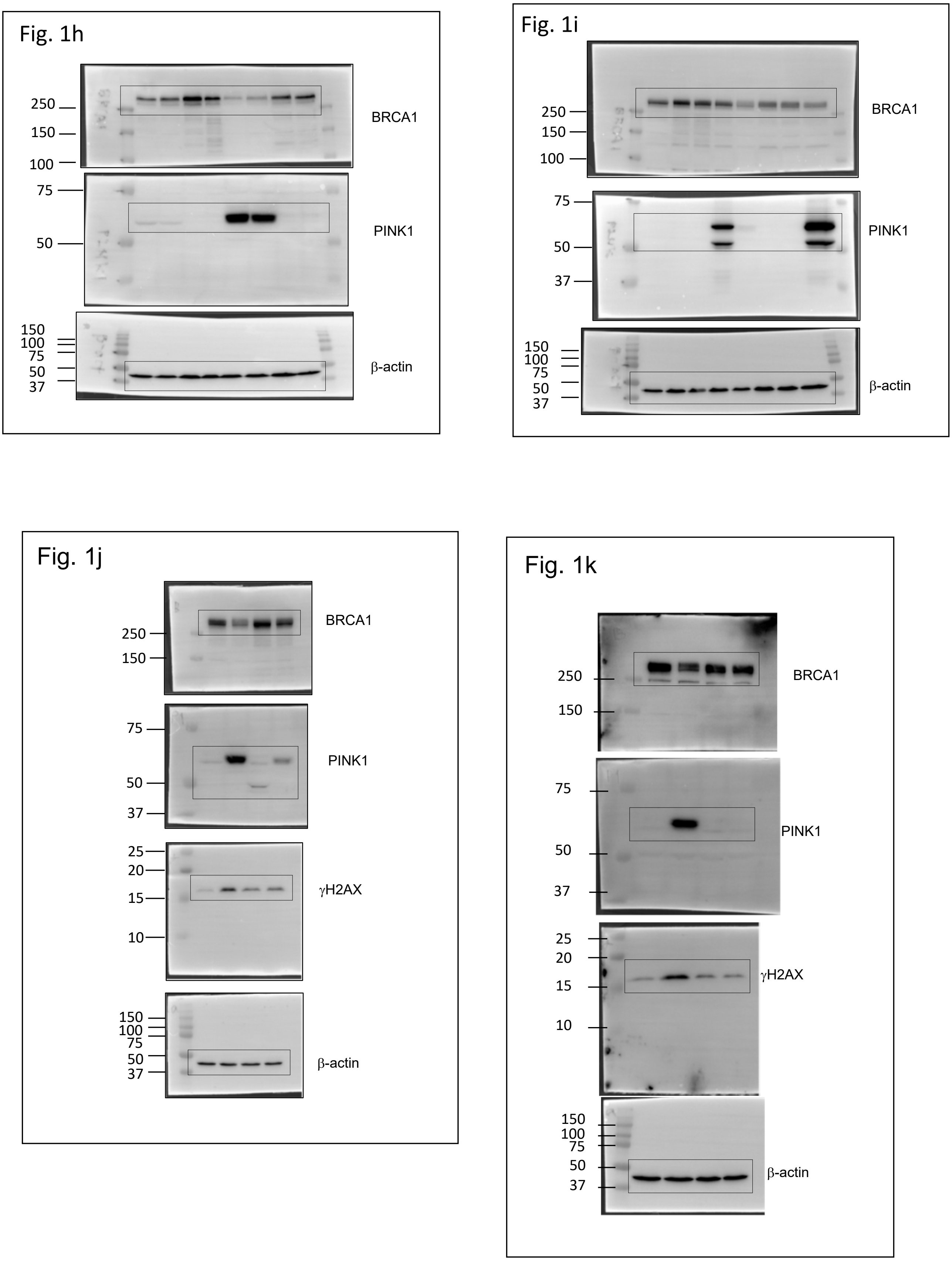

Figure S10. Original immunoblot images merged with corresponding marker images.

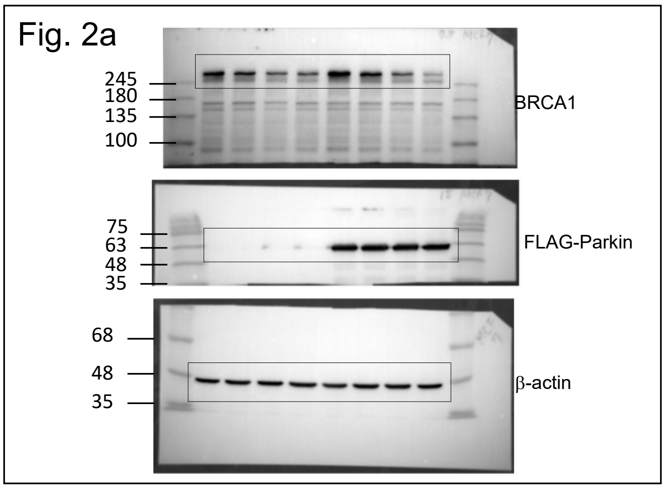

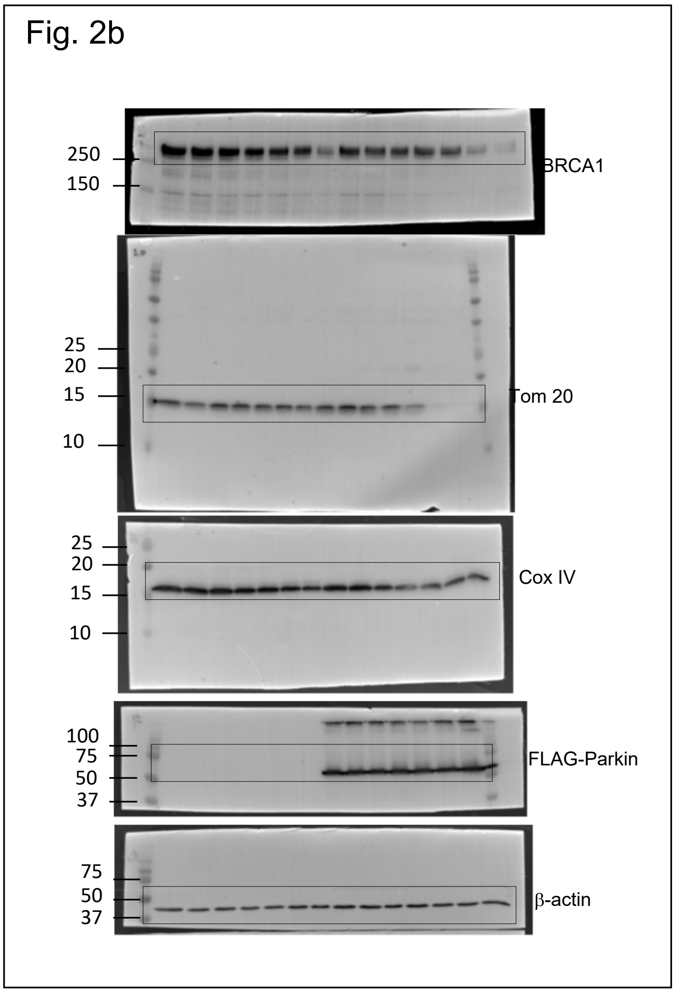

Figure S11. Original immunoblot images merged with corresponding marker images.

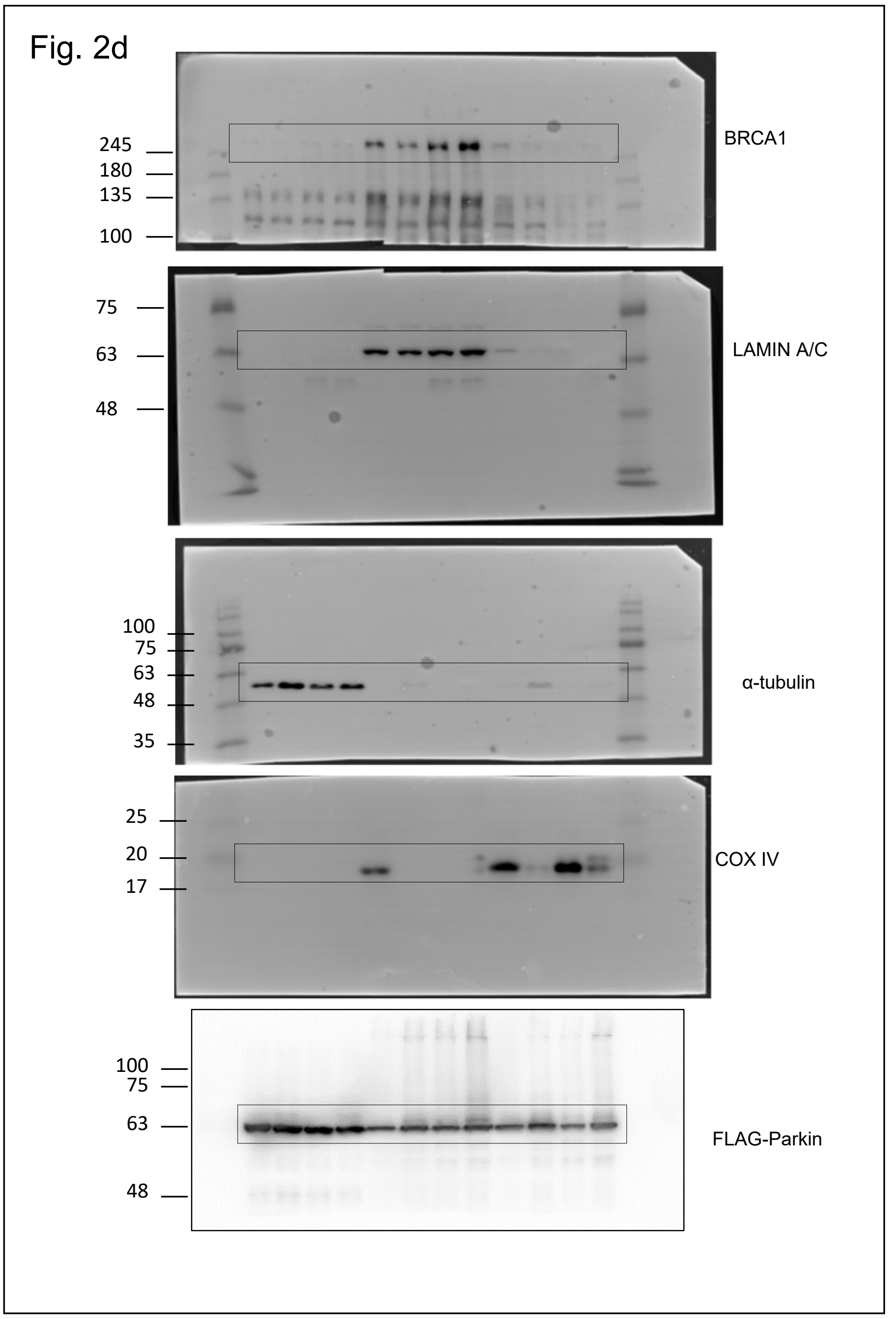

Figure S12. Original immunoblot images merged with corresponding marker images.

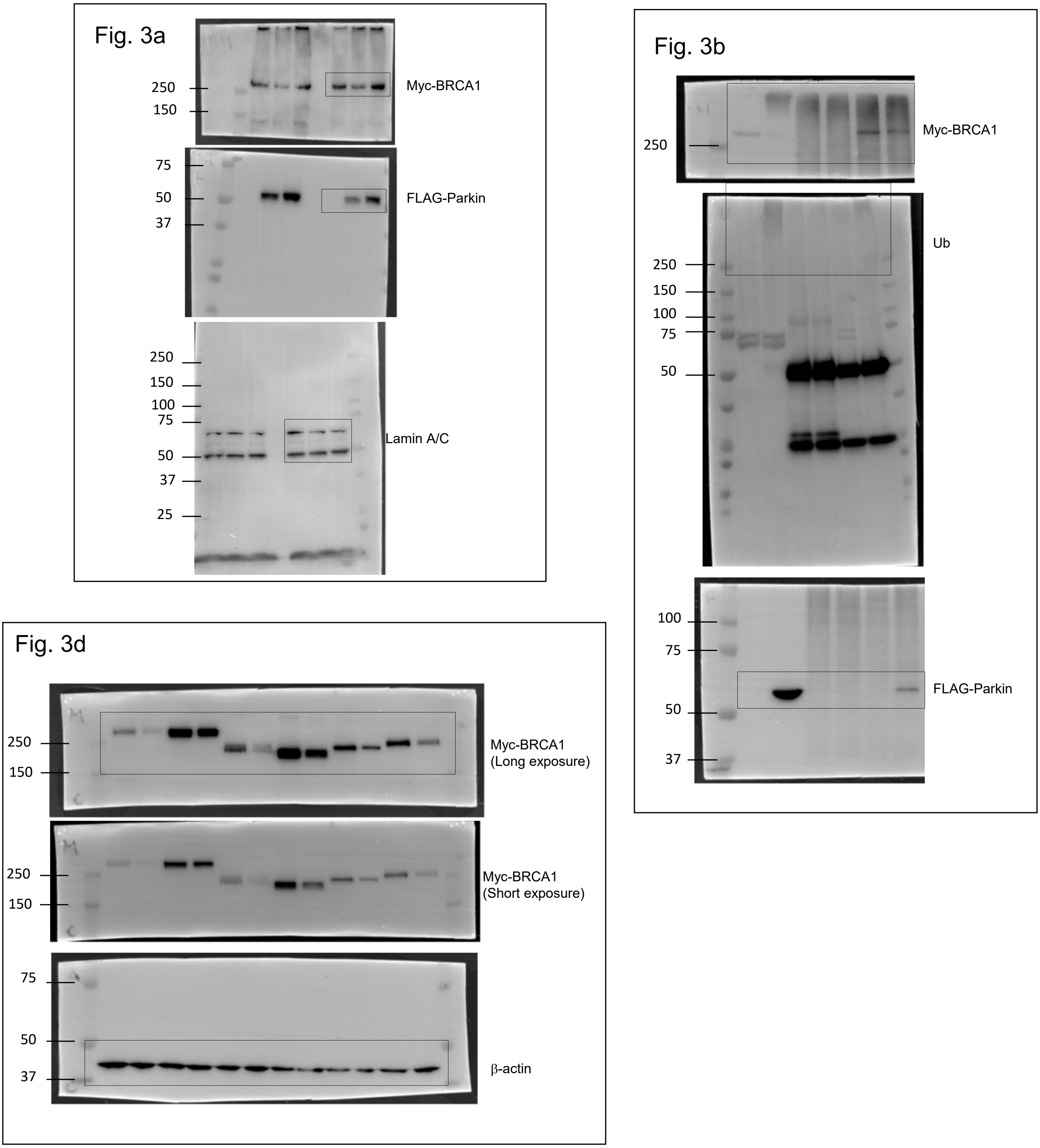

Figure S13. Original immunoblot images merged with corresponding marker images.

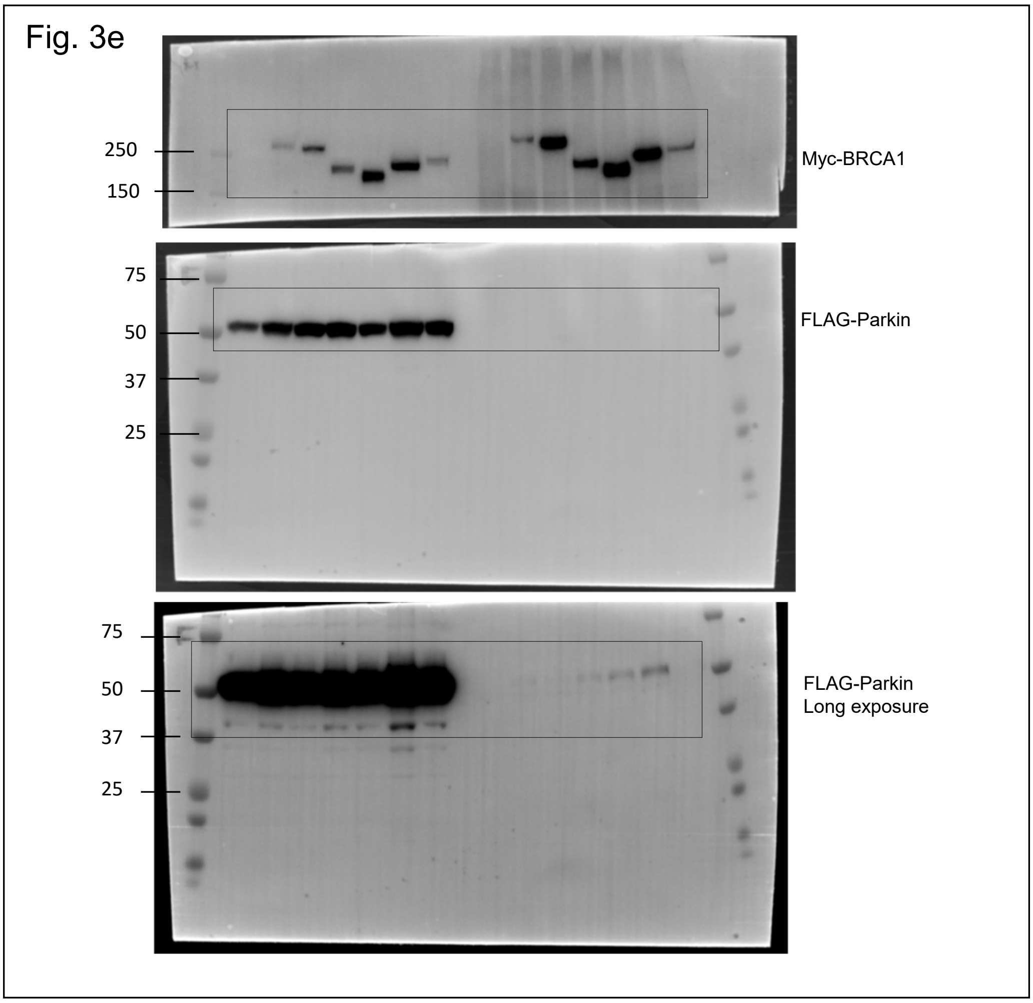

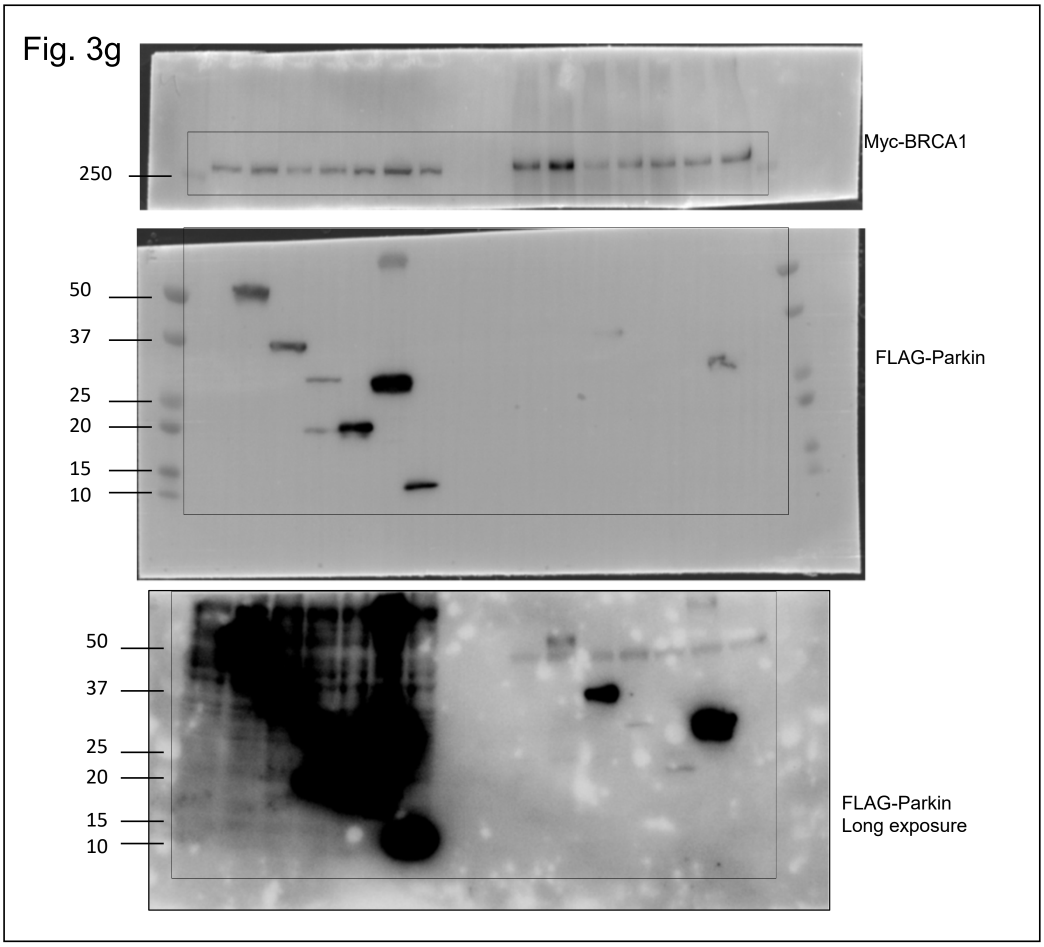

Figure S14. Original immunoblot images merged with corresponding marker images.

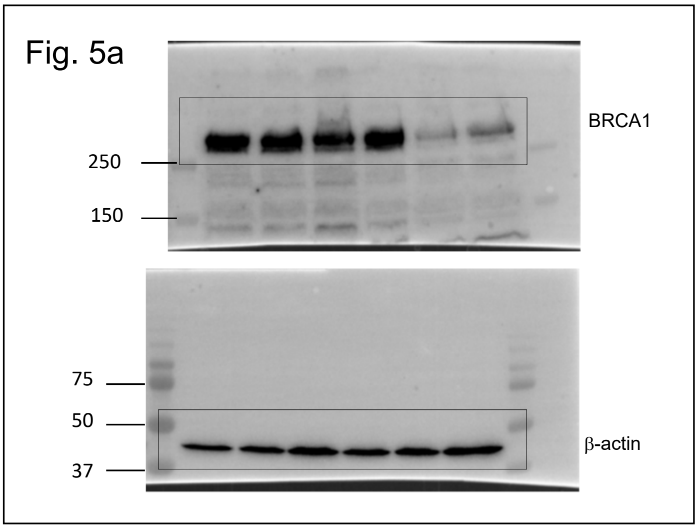

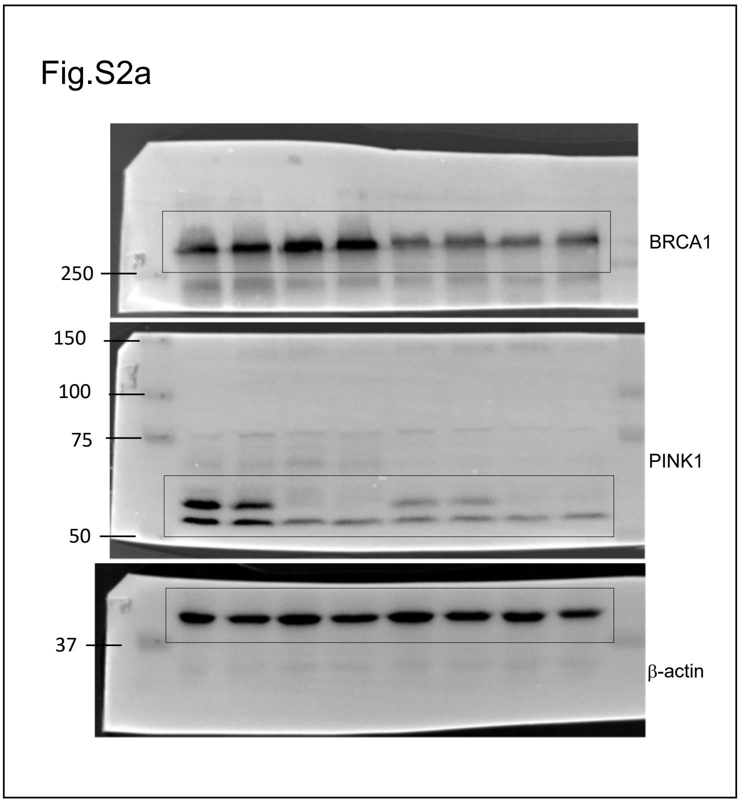

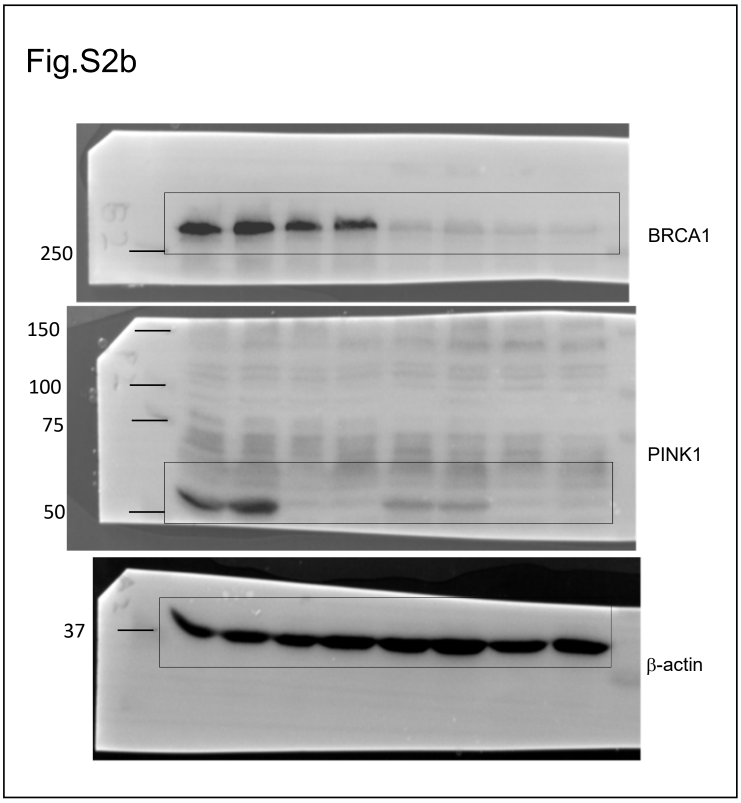

Figure S15. Original immunoblot images merged with corresponding marker images.

Fig.S3a

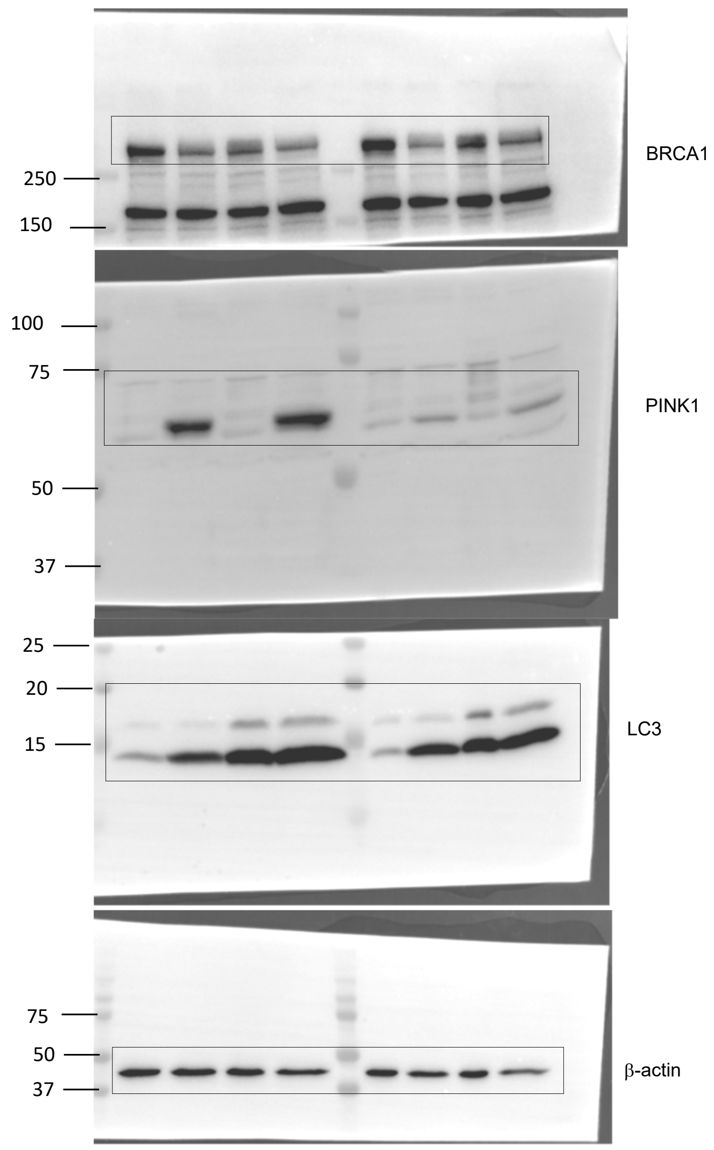

Fig.S3b

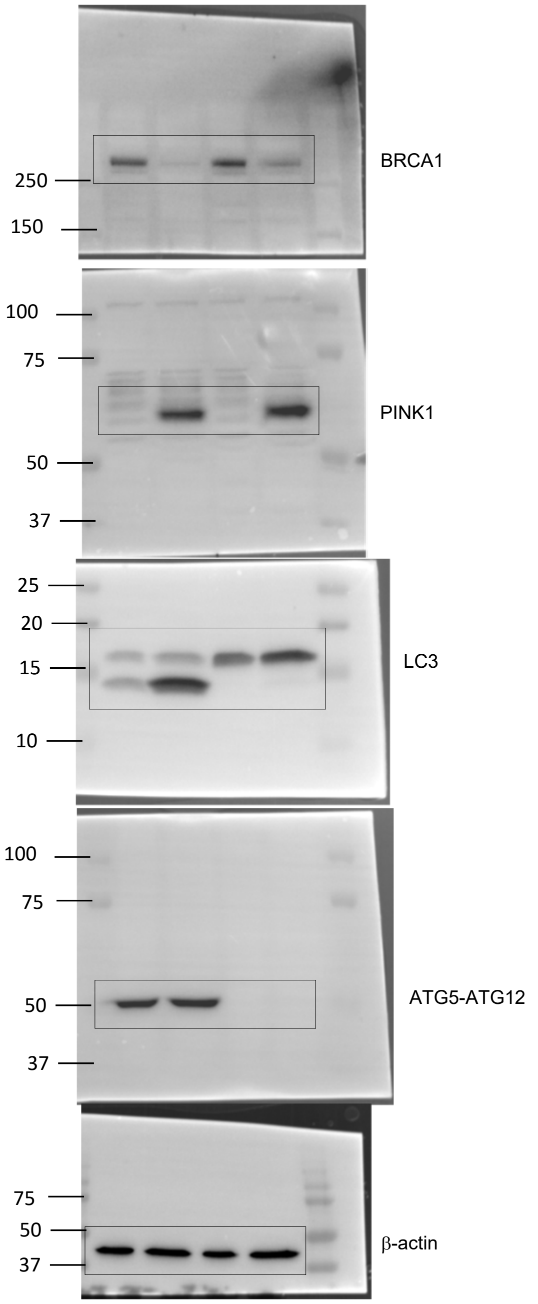

Figure S16. Original immunoblot images merged with corresponding marker images.

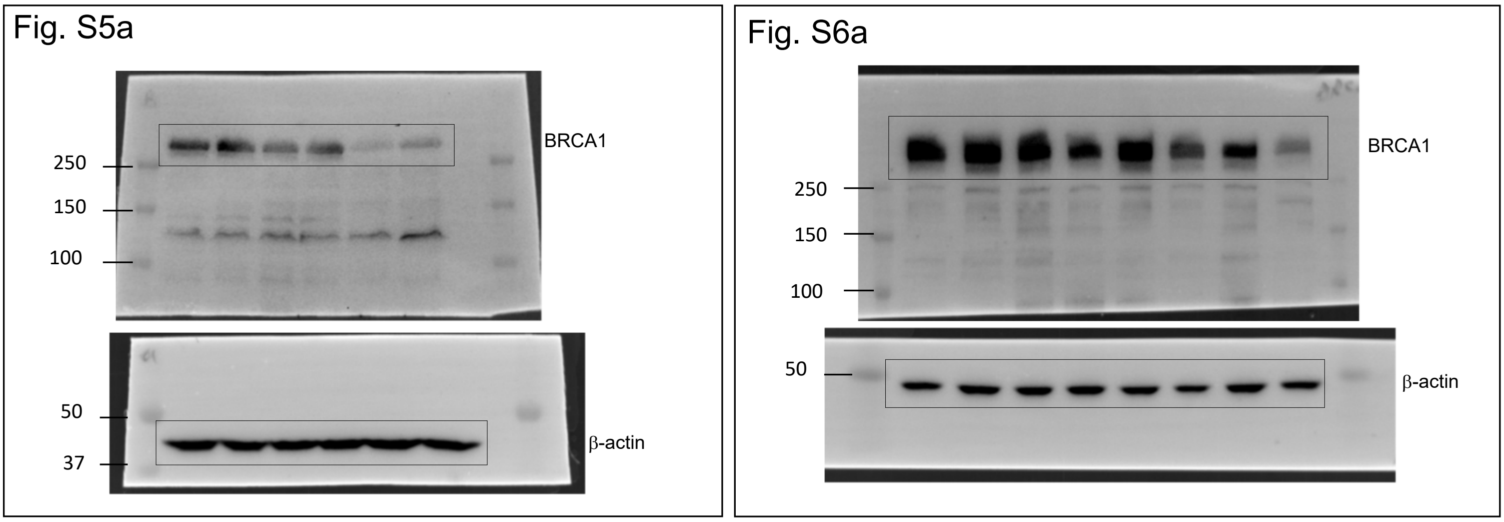

Figure S17. Original immunoblot images merged with corresponding marker images.
